# Exosome reporter mice reveal the involvement of exosomes in mediating neuron to astroglia communication in the CNS

**DOI:** 10.1038/s41467-019-11534-w

**Published:** 2019-09-12

**Authors:** Yuqin Men, Julia Yelick, Shijie Jin, Yang Tian, Ming Sum R. Chiang, Haruki Higashimori, Eoin Brown, Rachel Jarvis, Yongjie Yang

**Affiliations:** 1 Tufts University School of Medicine, Department of Neuroscience, 136 Harrison Avenue, Boston, MA 02111 USA; 2 Tufts University, Sackler School of Biomedical Sciences, 136 Harrison Avenue, Boston, MA 02111 USA; 3 Dongfang Hospital of University of Chinese Medicine, No.6, District 1, Fangxingyuan, Fangzhuang, Fengtai District, 100078 Beijing, People’s Republic of China

**Keywords:** Cell biology, Neuroscience, Astrocyte

## Abstract

Astroglia play active and diverse roles in modulating neuronal/synaptic functions in the CNS. How these astroglial functions are regulated, especially by neuronal signals, remains largely unknown. Exosomes, a major type of extracellular vesicles (EVs) that originate from endosomal intraluminal vesicles (ILVs), have emerged as a new intercellular communication process. By generating cell-type-specific ILVs/exosome reporter (CD63-GFP^f/f^) mice and immuno-EM/confocal image analysis, we found that neuronal CD63-GFP^+^ ILVs are primarily localized in soma and dendrites, but not in axonal terminals in vitro and in vivo. Secreted neuronal exosomes contain a subset of microRNAs (miRs) that is distinct from the miR profile of neurons. These miRs, especially the neuron-specific miR-124-3p, are potentially internalized into astrocytes. MiR-124-3p further up-regulates the predominant glutamate transporter GLT1 by suppressing GLT1-inhibiting miRs. Our findings suggest a previously undescribed neuronal exosomal miR-mediated genetic regulation of astrocyte functions, potentially opening a new frontier in understanding CNS intercellular communication.

## Abstract (web summary)

Our current understanding of exosome signaling among CNS cells is mostly limited to culture models. In this study, authors generated a new cell-type specific exosome reporter mouse line which allows the first in vivo investigation of the localization of neuronal exosomes in the CNS, and also potentially highlights the role of exosomally transferred miR-124-3p in mediating astroglial glutamate uptake function

## Introduction

Neuron to (astro)glia communication is essential for functional synaptic transmission and physiology in the Central Nervous System (CNS)^1,2^. Despite the important modulatory roles of astroglia in synapse function, molecular pathways that regulate the neuron-astroglia functional unit are largely undefined. During development, neuron-derived ligands (for example, JAG1, DLL1, and recently neurexins) interact with astroglial Notch^3^ or neuroligins^4^ to control astrocyte differentiation and morphogenesis^5^. Neuronal glutamatergic activity has also been implicated in regulating expression of important astroglial synaptic proteins, such as the predominant glutamate transporter GLT1^6,7^ and connexins^8^. In the adult brain, astrocytes are strategically localized near neuronal synapses to sense changes in neuronal activity, mainly through their surface receptors to pre-synaptically released neurotransmitters^9^, and respond by peri-synaptic morphological changes and release of signals (a process termed gliotransmission) to actively modulate synaptic transmission^10,11^.

Exosomes (50–150 nm in diameter), a major type of secreted extracellular vesicles (EVs)^12^, are derived from intraluminal vesicles (ILVs) in the early endosomal compartment and are released from cells during endosome maturation^13^. EVs and exosomes secreted from various CNS cell types have emerged as a novel and important intercellular communication pathway in the CNS^14^. We previously showed that cortical neurons secrete miR-124-containing exosomes that upregulate astroglial GLT1 expression in culture^15^. Hippocampal neurons also secrete EVs that facilitate the transfer of *arc* messenger RNA (mRNA) into recipient neurons and participate in activity-dependent translation^16^. EV-mediated intercellular signaling has also been found in invertebrate model organisms, as evidenced by the transfer of Wingless (*Wg*) from synaptic boutons to the specialized muscle region sub synaptic reticulum (SSR) in the neuromuscular junction (NMJ) of fly larva^17,18^. Exosomal signaling has also been implicated in pathological conditions of the CNS, including neurological injury^19^, neurodegenerative diseases^20^, and glioblastoma^21^.

MicroRNAs (miRs) are a class of noncoding RNAs with a length of 20–25 nucleotides that actively and significantly modulate development and disease processes in major organ systems, including the CNS. Although miRs typically modulate gene functions in cells where they are produced, miRs are often found in EVs, particularly exosomes, to shuttle between cells for intercellular signaling^22^. Intercellular transfers of miRs have been observed in CNS cells to regulate glutamate transporter function^15^, promote myelination^23^, and maintain brain vascular integrity^24^. EV-associated miRs also have potential as novel biomarkers for a range of neurological diseases^25^. It is important to note that the current understanding of exosome signaling among CNS cells is mostly limited to culture models. The specific cargos inside secreted EVs/exosomes from CNS cells are just beginning to be understood. Here, we developed a novel mouse model to illustrate intracellular ILVs and extracellular exosomes in vivo and in vitro. We identified miR cargos in secreted neuronal exosomes and determined how they exert non-cell autonomous genetic regulation following their transfer into astrocytes.

## Results

### Illustration of neuronal exosomes using exosome reporter mice

**Fig. 1:**
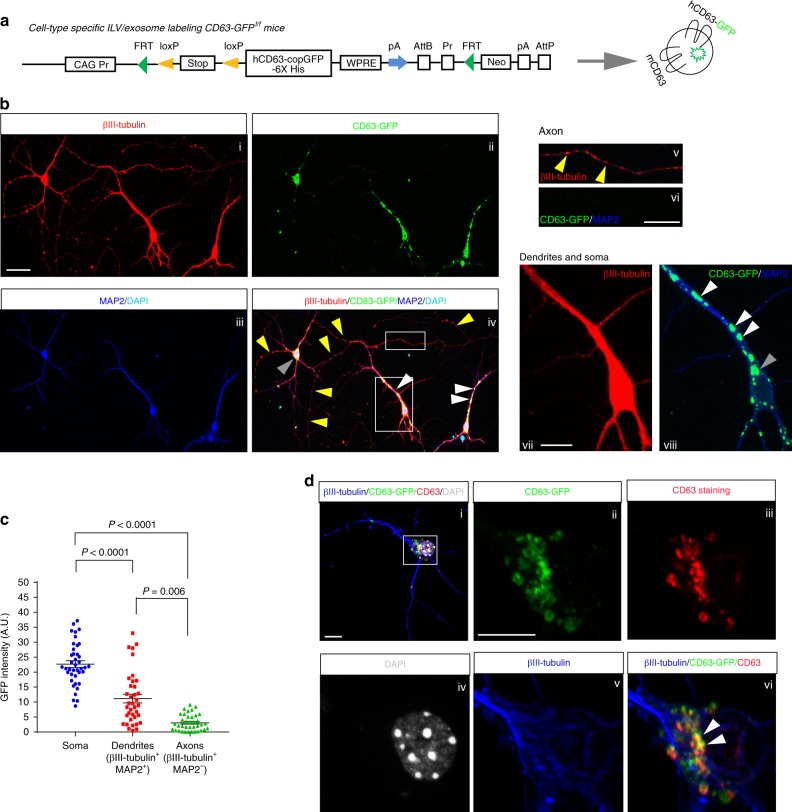
Compartmental localization of CD63-GFP^+^ ILVs in CD63-GFP^f/+^ neurons. **a** Schematic diagram of the gene targeting strategy to insert the CD63-copGFP-6xHis cassette into the *Rosa26* locus, in the intron between endogenous exons 1 and 2, for the generation of exosome reporter CD63-GFP^f/f^ mice. copGFP: GFP cloned from copepod *Pontellina plumata*; h or mCD63: human or mouse CD63; **b** Representative images of the distribution of CD63-GFP^+^ puncta in specific compartments of cultured primary neurons. Subpanels i: βIII-tubulin staining; ii: CD63-GFP fluorescence; iii: MAP2 and DAPI staining; vi: merge of CD63-GFP fluorescence and MAP2 staining; vii: magnified view of βIII-tubulin staining. Yellow arrows (subpanels iv and v): axons (βIII-tubulin^+^MAP2^−^) with sparse overlap of CD63-GFP^+^ puncta; White arrows (subpanels iv and viii): dendrites (βIII-tubulin^+^MAP2^+^) with abundant CD63-GFP^+^ puncta; Gray arrows (subpanels iv and viii): CD63-GFP^+^ puncta in the soma; Scale bar: 50 μm; v–viii, magnified confocal images of axons and dendrites taken with the 63x objective lens; **c** Quantification of CD63-GFP intensity in the soma, dendrites (βIII-tubulin^+^ MAP2^+^), and axons (βIII-tubulin^+^ MAP2^−^) of cultured primary neurons. *n* = 40–41 neurons/group. *P-*value was calculated using one-way ANOVA followed by a Tukey post-hoc test. **d** Representative images of CD63-GFP with endogenous mouse CD63 immunoreactivity. Scale bar: 10 μm; An anti-CD63 antibody specifically recognizing mouse CD63 was used. The magnified view of the CD63-GFP^+^ neuron (subpanel i) with each channel (as indicated near the image) is shown in ii–vi, respectively. white arrows: overlapped CD63-GFP with endogenous CD63 immunoreactivity

Several tetraspanin family proteins (CD81, CD9, and CD63) have been previously identified and validated as specific EV/exosome markers^26^. In particular, the tetraspanin CD63 has minimal expression on the plasma membrane, preferentially labels intracellular endosomal intraluminal vesicles (ILVs, precursor for exosomes) and secreted extracellular exosomes^26^. Despite the widely observed secretion of exosomes from multiple CNS cell types in vitro, exosome signaling in situ in the CNS remains essentially unexplored. We attempted the immunostaining of several tetraspanins (CD81 and CD9) and other exosome markers (Tsg101, Alix)^13,26^ on brain sections, which all give ambiguous immunostaining signals (data not shown). To explore in situ cell-type-specific ILV/exosome labeling in the CNS, we generated Cre-dependent ILV/exosome reporter (CD63-GFP^f/f^) mice by inserting a loxP-floxed stop codon upstream of human (h) CD63 tagged with copGFP (a GFP variant) and His-tag at its C-terminal (Fig. 1a). Human and mouse CD63 are highly conserved, sharing 88% of amino acid sequence, based on sequence alignment (data not shown). The homozygous CD63-GFP^f/f^ and heterozygous CD63-GFP^f/+^ mice can be reliably identified from PCR-based genotyping (Supplementary Fig. 1a).

We first explored the compartmental and subcellular localization of CD63-GFP in primary cortical neuronal cultures from CD63-GFP^f/+^ mice. AAV8-CaMKII-Cre virus was added, with optimization, into cultures to induce clear expression of CD63-GFP without inducing adverse effects on the survival and properties of neurons (Supplementary Fig. 1b). Interestingly, CD63-GFP fluorescence induced in CD63-GFP^f/+^ neurons is primarily localized peri-nuclear in neuronal soma (gray arrows, Fig. 1b, iv, viii, ~ 62%, Fig. 1c) and along βIII-tubulin^+^MAP2^+^ dendrites (white arrows, Fig. 1b, iv, viii, ~ 28%, Fig. 1c), but is sparse (~ 10%, Fig. 1c) along βIII-tubulin^+^ MAP2^-^ axons (yellow arrows, Fig. 1b, iv, v, additional examples in Supplementary Fig. 1c), by examining the co-localization of CD63-GFP with βIII-tubulin (total neurites) and MAP2 (dendrite-specific) immunoreactivity under magnified views (63x objective lens) of axons (Fig. 1b, v–vi) or dendrites (Fig. 1b, vii–viii). We quantified GFP fluorescence intensity along the total length (proximal and distal) of axons. The limited labeling of CD63-GFP along axons remains unchanged even with a higher titer AAV8 and longer incubation time (data not shown). CD63-GFP fluorescence is also co-localized with Rab7 (MVB/late endosome marker) immunoreactivity in neuronal soma (Supplementary Fig. 1d), confirming its subcellular localization with endosomes, the origin of ILVs and secreted exosomes, but not with the plasma membrane-shed microvesicles. Additionally, induced CD63-GFP overlaps well with endogenous CD63 immunoreactivity (white arrows, Fig. 1d, vi), indicating minimal abnormal accumulation and random expression of induced CD63-GFP in neurons. Expression of CD63-GFP can also be induced in primary glial cultures (astrocytes, microglia, oligodendrocytes) prepared from CD63-GFP^f/+^ mice using either AAV5-*gfap*-Cre (astrocytes) or AAV-*pgk*-Cre (microglia and oligodendrocytes) viruses (Supplementary Fig. 1e), confirming cell-type-specific labeling of CD63-GFP^+^ ILVs in CNS cell types and allowing the study of CD63-GFP^+^ ILVs/exosomes from glial cells in the future.

**Fig. 2:**
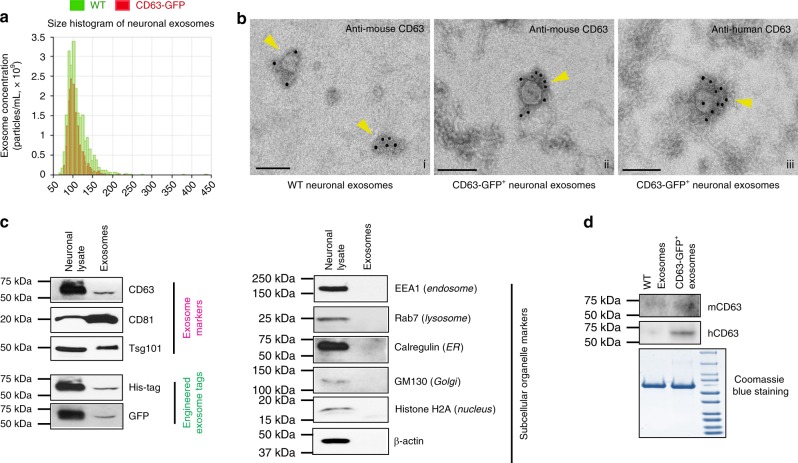
Secretion of CD63-GFP^+^ exosomes from CD63-GFP^f/+^ primary neurons. **a** Representative size distribution of exosomes isolated from conditioned medium of cultured WT (green) and AAV-CaMKII-Cre transduced CD63-GFP^f/+^ (red) primary neurons by the qNano particle analyzer. **b** Representative immuno-Electron Microscopy (EM) images of human and mouse CD63 labeling on in vitro prepared WT and CD63-GFP^+^neuronal exosomes. Scale bar: 100 nm; Subpanels i: anti-mouse CD63 on WT neuronal exosomes; ii: anti-mouse CD63 on CD63-GFP^+^ neuronal exosomes; iii: anti-human CD63 on CD63-GFP^+^ neuronal exosomes. Images were from a total of 40–50 images of two separate preparations. **c** Representative immunoblots of typical protein markers for exosomes, engineered exosome tags, and other subcellular organelles from lysate and purified exosome fractions of AAV-CaMKII-Cre transduced CD63-GFP^f/+^ neurons. **d** Representative immunoblot of induced human CD63 and endogenous mouse CD63 from WT and CD63-GFP^+^ neuronal exosomes. Antibodies specifically recognizing either human or mouse CD63 were used

To determine whether the expression of CD63-GFP alters overall exosome secretion, we analyzed the size and quantity of extracellular vesicles isolated from neuron conditioned medium using the qNano particle analyzer^27^ and found a single Gaussian peak at a mean of 91 nm (62 to 164 nm range, Fig. 2a), typical of in vitro prepared exosomes in both WT (in green) and CD63-GFP^+^ (in red) neuronal exosomes. The total number of secreted WT (mean quantity: 2.26 × 10^12^ particles/mL) and CD63-GFP^+^ (mean quantity: 2.1 × 10^12^ particles/mL) exosomes is comparable with a modest decrease (8%) in CD63-GFP^+^ samples. The size distribution of these exosomes is consistent with our previous EM analysis of neuronal culture-derived exosomes^15^. Subsequent immunogold labeling of either mouse or human CD63 on WT and CD63-GFP^+^ neuronal exosomes also showed similar size and comparable mouse CD63 labeling on these exosomes (Fig. 2b, i–ii). The presence of typical exosomal marker proteins, the tetraspanins CD63 and CD81, and a member of the ESCRT-I complex Tsg101 (Fig. 2c) was detected in the vesicular fraction isolated from neuron conditioned medium following serial centrifugation steps. These markers were not detected in exosome-free supernatant (data not shown). There is also no contamination by cytoplasmic organelles or apoptotic bodies, indicated by undetectable EEA1, Calreticulin, GM130, Rab7, and Histone H2A, in the vesicular fraction (Fig. 2c). Meanwhile, engineered exosomal GFP and His-tag were also detected, at the same molecular weight (60 KDa) as CD63 (Fig. 2c), in the vesicular fraction and their expression levels are 10% (GFP) or 17% (His-tag) of their levels in neuronal lysate, providing a quantitative assessment of the proportion of ILVs that are secreted as exosomes from primary neurons. This 10–17% is comparable to the relative expression levels of CD63 in the exosome fraction vs. neuronal lysate (15%).

The expression of CD63-GFP has no effect on other tetraspanins, such as CD81 (Supplementary Fig. 2a). We also compared the relative expression levels of induced human (h) and endogenous mouse (m) CD63 in WT and CD63-GFP^+^ neurons and their secreted exosomes using immunoblot with specific anti-CD63 antibodies that detect only human or mouse CD63. The mCD63 expression levels in CD63-GFP^+^ neuronal lysate and exosomes are equivalent to the mCD63 levels in WT neuronal lysate (Supplementary Fig. 2b) and exosomes (Fig. 2d) while the hCD63 is only detected in CD63-GFP^+^ neuronal lysate and exosomes (Supplementary Fig. 2b and Fig. 2d). Interestingly, exosomal proteins are mostly concentrated in the 50–75 KDa range, revealed by Coomassie blue staining, indicating a potentially unique composition of exosomal proteins. In addition, mCD63 expression levels in CaMKII-CreER^+^CD63-GFP^+^ brain (cortex) lysate are also comparable to that of WT brain lysate (Supplementary Fig. 2c), further confirming that the induction of hCD63 has minimal effects on the expression of endogenous mCD63. Overall, these immunoblotting and immuno-EM results also suggest that CD63-GFP^+^ ILVs can faithfully traffic intracellularly and be secreted extracellularly as endogenous CD63^+^ ILVs.

**Fig. 3:**
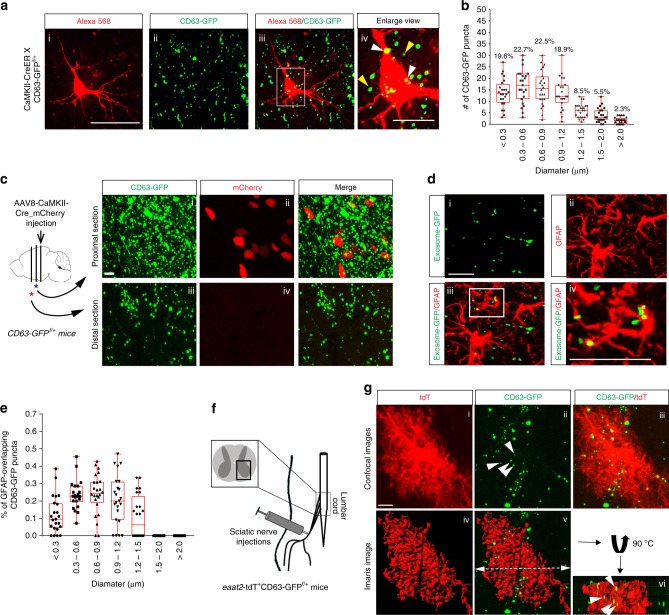
In situ illustration of neuron-secreted exosomes internalized into astrocytes. Representative confocal images (**a**) and quantification (**b**) of the number and size of CD63-GFP^+^ puncta in cortical slices of CaMKII-CreER^+^CD63-GFP^f/+^ mice. Neurons were patched and filled with Alexa-568 dye Subpanels i: Alexa-568 dye filled neuron; ii: CD63-GFP fluorescence: iii: merge of Alexa-568 dye filled neuron and CD63-GFP; iv: magnified view of iii. A single dose of 4-OHT (10 mg/kg) was given to P15-16 mice. Scale bar: 50 μm (i–iii); 15 μm (enlarged view, iv); *n* = 24 images/4 mice; **c** Representative images of induced CD63-GFP^+^ puncta in proximal and distal sections to the injection site. Subpanels i, iii: CD63-GFP fluorescence; ii, iv: mCherry expression from AAV8-CaMKII-Cre_mCherry virus; The virus injection site and brain sections analyzed are illustrated in the diagram. Red star: distal sections; blue star: proximal sections; Scale bar: 40 μm; **d** Representative confocal images of the internalization of neuron-secreted exosomes (CD63-GFP^+^ puncta) into GFAP^+^ astrocytes in AAV8-CaMKII-Cre_mCherry virus injected CD63-GFP^f/+^ mice. Subpanels i: CD63-GFP fluorescence; ii: GFAP immunostaining: iii: merge of CD63-GFP and GFAP staining; iv: magnified view of iii; Scale bar: 50 μm (i–iv); **e** Quantification of the size and number of CD63-GFP^+^ puncta that overlap with GFAP^+^ astrocytes. *n* = 24 images/4 mice; **f** Diagram of peripheral sciatic nerve injections of AAV9-CaMKII-Cre/FluoroGold to *eaat2*-tdT^+^CD63-GFP^f/+^ mice. **g** Representative confocal and Imaris 3D images for astroglial localization of neuron-derived CD63-GFP^+^ exosomes. Subpanels i: tdT^+^ astrocyte; ii: CD63-GFP fluorescence; iii: merge of CD63-GFP and tdT fluorescence; iv: converted 3D astrocyte domain from (i); v: merge of CD63-GFP and converted 3D astrocyte domain; vi: the upper part of the transected image in (v) with 90 degree outward rotation; Scale bar: 10 μm; The dashed line indicates where the transection was. The upper part of the transected image turns 90 degree outward to show the representative CD63-GFP^+^ puncta (white arrows) inside tdT^+^ astrocytes. The data was presented in the box and whisker plot with defined elements, median (center line), upper and lower quartiles (bounds of box), and highest and lowest values (whiskers)

How neuronal ILVs/exosomes are localized and distributed in situ in the CNS is currently unknown. Encouraged by the ILV/exosome labeling of CD63-GFP in primary neurons, we next examined in situ neuronal CD63-GFP signals in cortical sections of CaMKII-CreER^+^CD63-GFP^f/+^ mice. A single injection of 4-OHT (intraperitoneal (i.p.), 10 mg/kg) induces clear expression of CD63-GFP, primarily from excitatory neurons based on the specificity of the CaMKII promoter (typical neuronal soma and neurites, Supplementary Fig. 3a), in the brain and spinal cord (Supplementary Fig. 3b). 4-OHT-injected CaMKII-CreER^+^CD63-GFP^f/+^ mice appear normal and the induced CD63-GFP signals are largely overlapped with endogenous CD63 immunoreactivity in motor cortex (80%), hippocampus CA1 (100%), and striatum caudate putamen (94%) (white arrows, Supplementary Fig. 3c, iii, vi, and ix), confirming its same localization as the endogenous CD63 protein on brain sections. It is noted, however, that endogenous CD63 immunostaining signals only partially and differentially overlap with CD63-GFP in these brain regions (motor cortex: 40%; hippocampal CA1: 80%; and striatum Cpu: 15%). This is expected, as endogenous CD63 staining includes both neuronal and glial staining signals. On the other hand, CD63-GFP is only induced in neurons in CaMKII-CreER^+^CD63-GFP^f/+^ mice and neurons are typically more abundant in hippocampal CA1than in cortex and even more so than in striatum. Magnified confocal images further revealed abundant, individual CD63-GFP^+^ puncta (Fig. 3a), some of which are co-localized with the soma of Alexa-568-filled cortical pyramidal neurons (white arrows, Fig. 3a, iv). The size distribution of CD63-GFP^+^ puncta ranges from less than 0.3 μm (19.6%) to greater than 2 μm (2.3%), as shown in Fig. 3b. As CD63^+^ ILVs are derived from endosome and CD63 is also known to label lysosome inside the cell, it is likely that the larger CD63-GFP^+^ puncta label these subcellular organelles and are therefore intracellularly localized. Although the overall size distribution of the majority of CD63-GFP^+^ puncta signals are significantly larger than the typical size (50–150 nm) of exosomes prepared from cultures, this could be due to the filtration procedure used (0.22 μm filter) in preparing in vitro exosomes and resolution limit of typical confocal microscopy, which is unable to resolve smaller puncta.

To determine whether neuronal CD63-GFP^+^ signals are able to migrate from the site of induction as extracellular exosomes in vivo, AAV8-CaMKII-Cre_mCherry virus particles (0.5 μL, 1 × 10^12^ particles/mL) from which mCherry-tagged Cre is expressed under the CaMKII promoter were focally injected into the motor cortex of CD63-GFP^f/+^ mice (Fig. 3c diagram). Both CD63-GFP and mCherry signals are observed in the proximal ( < 100 μm) section from the injection site (Fig. 3c, i–ii), indicating effective AAV8-mediated Cre expression and CD63-GFP induction from the injection. Interestingly, CD63-GFP signals are also observed in the distal (~ 500 μm) section (Fig. 3c, iii) from the injection site where there is no mCherry fluorescence (Fig. 3c, iv), suggesting that these CD63-GFP signals were not locally induced, but migrated from the injection site. Although the CaMKII promoter selectively induces CD63-GFP expression from excitatory neurons, CD63-GFP signals are found to co-localize with GFAP immunoreactivity (white arrows, Fig. 3d) at the distal section, indicating that these CD63-GFP^+^ puncta are potentially transferred to astrocytes after they are induced from the original AAV8-transduced excitatory neurons. Quantitative analysis found that 10–22% of the CD63-GFP^+^ puncta are co-localized with GFAP immunostaining signals, depending on the size of co-localized CD63-GFP puncta (Fig. 3e), suggesting that extracellular CD63-GFP^+^ puncta signals, presumably secreted neuronal exosomes, are at least 10–22% of total CD63-GFP^+^ puncta signals. This is also consistent with the relative GFP or His-tag expression levels (10–20%) in neuronal exosomes vs. neuronal lysate from primary neuronal cultures (Fig. 2c).

To further demonstrate that neuron-secreted exosomes are internalized into astrocytes, we utilized the peripheral sciatic nerve projections of spinal motor neurons and injected a mixture of AAV9-CaMKII-Cre and FluoroGold (FG) dye into sciatic nerves of *eaat2*-tdT^+^CD63-GFP^f/+^ double-positive mice, as shown in Supplementary Fig. 3d, i, so that AAV9-CaMKII-Cre and FG can be retrograde co-transported only to spinal motor neurons (SMNs) and induce expression of CD63-GFP in FG^+^ SMNs (Supplementary Fig. 3d, ii–v). The selective delivery of FG/AAV9-CaMKII-Cre helps determine the source of secreted CD63-GFP^+^ exosomes from FG^+^ SMNs. Meanwhile, we also observed CD63-GFP^+^ puncta scattered around FG^+^ spinal MNs (Supplementary Fig. 3d, ii). Some of which are co-localized with tdT labeled astrocytes of *eaat2*-tdT^+^ mice (Fig. 3g, i–iii). Careful analysis of astrocyte three-dimensional (3D) domain built using Imaris software (Fig. 3g, iv) and the transection view of astrocyte domain (Fig. 3g, v, vi) clearly illustrate the localization of CD63-GFP^+^ puncta inside tdT^+^ astrocytes, further demonstrating that neuronal exosomes can be secreted and transferred into astrocytes in vivo.

**Fig. 4:**
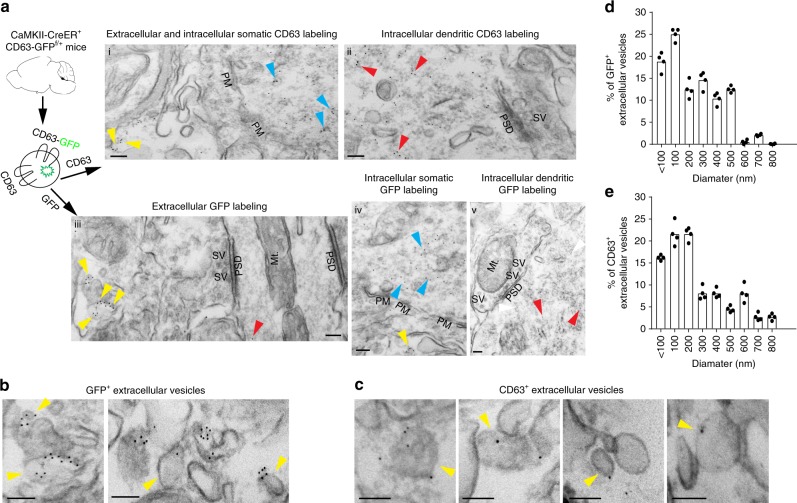
In situ localization of neuronal ILVs and exosomes revealed by immuno-EM. **a** Representative immuno-EM of CD63 and GFP, respectively, in the brain of CaMKII-CreER^+^CD63^f/+^ mice. Immunogold labeling of CD63 and GFP were separately performed on the same CaMKII-CreER^+^CD63^f/+^ mouse brain. Subpanels i: extracellular and intracellular somatic CD63 labeling; ii: intracellular dendritic CD63 labeling; iii: extracellular GFP labeling; iv: intracellular somatic GFP labeling; v: intracellular dendritic GFP labeling; Blue arrows: intracellularly localized CD63 or GFP puncta; Yellow arrows: extracellularly localized CD63 or GFP puncta; Red arrows: Dendritically localized CD63 or GFP puncta; PM: plasma membrane; SV: synaptic vesicles; PSD: post-synaptic density; Mt: mitochondria; Scale bar = 100 nm. Representative GFP^+^ (**b**) or CD63^+^ (**c**) extracellular vesicles from CaMKII-CreER^+^CD63^f/+^ brain sections (cortex and hippocampus). Yellow arrows: extracellularly localized CD63^+^ or GFP^+^ vesicles; Scale bar: 100 nm; Quantification of extracellular GFP^+^ (**d**) or CD63^+^ (**e**) vesicles from CaMKII-CreER^+^CD63^f/+^ brain sections. The anti-CD63 antibody specifically recognizing human CD63 was used. *n* = 120–150 images from four mice; mean value from each mouse quantification was presented as individual data point

To overcome the detection limit of confocal microscopy, we further examined CD63 and GFP signals, respectively, by immuno-EM in brain sections of CaMKII-CreER^+^CD63-GFP^f/+^ mice. Similar to the distribution of CD63-GFP fluorescence observed in primary neurons (Fig. 1b, c), immunogold (IG) labeling particles (5 nm) of both CD63 and GFP from the same brain sample are commonly observed in either soma (blue arrows, Fig. 4a, i, iv) or dendrites (red arrows, Fig. 4a, ii, iii, and v), but not in axon terminals (Fig. 4a, ii, iii, and v) of cortical and hippocampal neurons. Soma, dendrites, and axon terminals are indicated by clear plasma membrane (PM) boundary, post-synaptic density (PSD), or synaptic vesicles (SV), respectively, from the immuno-EM images. The somatodendritic localization of CD63 and GFP IG labeling particles in neurons is consistent with the endosomal origin of ILVs. Extracellular CD63 and GFP IG particles are also frequently observed (yellow arrows, Fig. 4a, i, iii, and iv). Magnified GFP^+^ or CD63^+^ extracellular vesicular structures (Fig. 4b, c) are clearly visible from brain sections (cortex and hippocampus) and further support the secretion of exosomal vesicular structures in vivo in the brain. Quantification of extra- and intracellular CD63 and GFP IG puncta found that 10–15% of CD63 or GFP IG particles are extracellularly localized, comparable to confocal image-based quantification of extracellular CD63-GFP^+^ puncta signals (Fig. 3e). Specifically, 55–60% of GFP^+^ or CD63^+^ extracellular vesicles are no >200 nm (Fig. 4d, e), the typical size range of in vitro prepared exosomes. Interestingly, ~ 25% of GFP^+^ or CD63^+^ extracellular vesicles are between 300–500 nm in size (Fig. 4d, e), larger than the size of in vitro prepared exosomes. This could be due to the filtration step (0.22 μm) used for preparing exosomes from culture media or could represent an inherent difference between in vivo and in vitro neuronal exosomes. The size and labeling of CD63-GFP on extracellular vesicles is also comparable to the size and labeling of endogenous mouse CD63^+^ extracellular vesicles, indicated by multiple examples of endogenous mouse CD63 labeling immuno-EM images (Supplementary Fig. 3e). As GFP is tagged to CD63 and selectively induced in 4-OHT-injected CaMKII-CreER^+^CD63-GFP^f/+^ mice, immuno-EM analysis of both CD63 and GFP and their recurrent extracellular localization confirmed our confocal image-based observation that exosomes/EVs can be commonly secreted from neurons in situ in the brain.

### Identification of miR cargos in neuronal exosomes

**Fig. 5:**
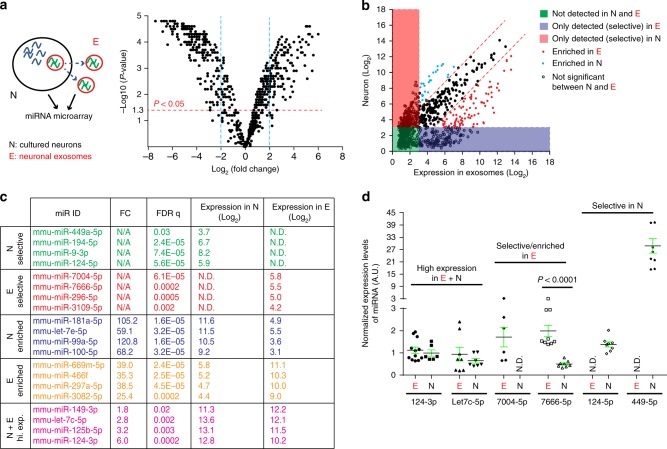
Neuronal exosomes contain an abundant and selective subset of miRs. **a** Schematic diagram and volcano plot of differentially expressed neuronal and neuronal exosomal miRs identified by miR microarray hybridization. *n* = 3 biologically independent samples per condition. For each independent experiment, exosomes were prepared from same batch of primary neurons from which the total neuronal RNA was also prepared. N: neurons; E: neuronal exosomes; miRs with expression values < 2^3^ arbitrary unit (A.U.) from the microarray hybridization are deemed not expressed. **b** Identification of enriched or selective miRs contained in either neurons or secreted exosomes by miR microarray hybridization. Green shaded area: miRs not expressed in either neurons or exosomes ( < 2^3^ A.U. in both E and N); red shaded area: miRs selectively expressed in neurons ( < 2^3^ A.U. in E, > 2^3^ A.U. in N, FDR *q* < 0.05); purple shaded area: miRs selectively expressed in exosomes ( < 2^3^ A.U. in N, > 2^3^ A.U. in E, FDR *q* < 0.05); red dots: miRs enriched in E (FC_E/N_ > 4, *P* < 0.05); blue dots: miRs enriched in N (FC_E/N_ < -4, FDR *q* < 0.05); black dots: miRs not enriched in either N or E (FC < 4, FDR *q* < 0.05). **c** Representative selective or enriched miRs from both neurons and neuronal exosomes, respectively. N.D.: not detected; N/A: not applicable; **d**Validation of representative miRs that are neuron and exosome selective or enriched, respectively, by qPCR. All original Ct values are within 26–32 range, Ct > 34 is deemed not detected (N.D.). U6 small nuclear (sn) RNA was used as the endogenous control. *n* = 3–6 biologically independent samples (four technical replicates/sample) per condition. *P-*values were determined from two-tailed unpaired *t*-test. Error bars were presented in S.E.M

Studies from non-CNS cells have shown that both messenger and regulatory RNAs are commonly packed into exosomes and secreted to mediate various functions in recipient cells^22^. Although neuronal exosomal cargos remain largely unexplored, the presence of abundant mRNAs and regulatory RNAs especially microRNAs (miRs), has been suggested^15,28^. To identify abundant and selective exosomal miRs from neuronal miRs, we performed miR microarray hybridization using total RNA isolated from cultured neurons and their secreted exosomes (Fig. 5a diagram). As shown in the volcano plot (Fig. 5a), the miR profile in neuronal exosomes is drastically different from that in neurons, even though all miRs are initially transcribed and processed (Pri-miR to Pre-miR) in the neuronal nucleus. Interestingly, a subset of miRs is only detected in either exosomal (95, purple shade in Fig. 5b) or neuronal (168, red shade in Fig. 5b) samples. By using a fourfold difference in the expression level as the cutoff, we further found 47 (red dots, Fig. 5b) or 78 (blue dots, Fig. 5b) miRs enriched in exosomal or neuronal samples, respectively. Neuron selective or enriched miRs include miR-9-3p, -181a-5p, and -let-7e-5p (Fig. 5c) that were previously shown to be neuron-specific and are vital for neuronal identity^29–31^. In contrast, miRs such as miR-669, -466 family members, miR-297a-5p, and miR-3082-5p (Fig. 5c) are selectively present or enriched in neuronal exosomes. All selective or enriched miRs either in neurons or neuronal exosomes are summarized in Supplementary Tables 1–4.

Although previous studies have found that several miRs (miR-451, miR-150, and miR-142-3p) are preferentially packed in exosomes of non-CNS cells^22,32^, these miRs are rarely found in neuronal exosomal samples. This could be a result of an overall miR profile difference of neurons compared to non-CNS cells. It may also reflect the miR composition difference of neuronal exosomes vs. exosomes secreted from non-CNS cells. Both neuronal exosomes and neurons also share a number of highly expressed miRs, as shown in Fig. 5c (only the ones with Log_2_ > 10) and Supplementary Table 5. It is particularly interesting that miR-124-3p, one of the miRs essential for neuronal identity and functions^33^, is highly present in neuronal exosomes. As neuronal exosomes are secreted vesicles, high levels of exosomal miRs can potentially serve as a new intercellular signal to recipient cells, especially to glial cells for non-cell autonomous functions in these cells. It is particularly intriguing that only miR-124-3p, but not miR-124-5p, is sorted into neuronal exosomes, even though both are derived from the same primary transcript pri-miR-124. The sorting and packing mechanisms of miRs into neuronal exosomes are currently not well-understood. Representative miRs that are highly expressed in both neurons and neuronal exosomes (124-3p, Let-7c-5p), or selective/enriched in either neurons (124-5p, 449-5p) or neuronal exosomes (7004-5p, 7666-5p) were further confirmed in exosomal and neuronal samples using quantitative reverse transcriptase PCR (qRT-PCR) (Fig. 5d). The expression of CD63-GFP also has very minimal effects on the relative expression of these representative miRs in neurons or neuronal exosomes (Supplementary Fig. 4a).

### Elevated astroglial miR-124-3p involves neuronal exosomes

**Fig. 6:**
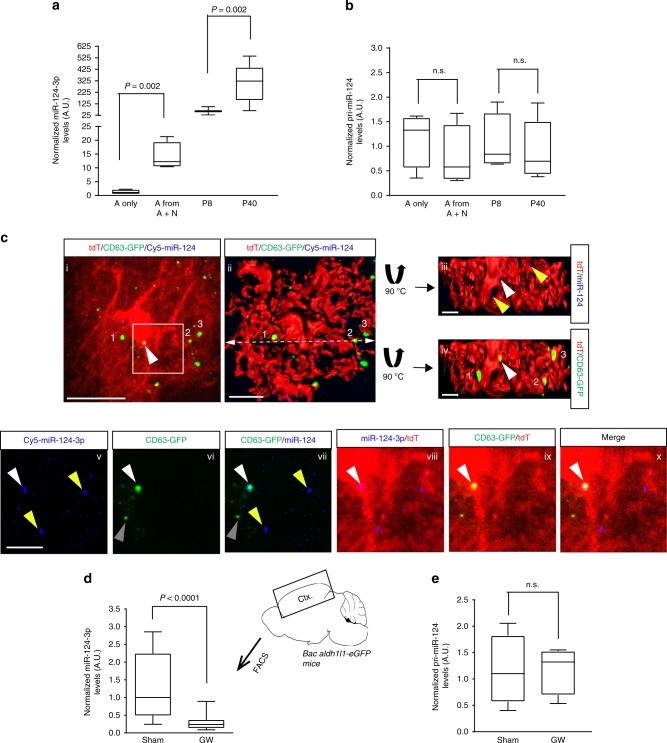
In vivo transfer of miR-124-3p into astrocytes through secreted exosomes. Expression levels of miR-124-3p (**a**) and pri-miR-124 (**b**) in cultured astrocytes (A only), astrocytes from astrocyte and neuron co-cultures (A from A + N), and astrocytes isolated from cortex by FAC sorting from Bac *aldh1l1*-eGFP mice at P8 (A from P8) or P40 (A from P40). *n* = 4–6 biologically independent samples per condition. *P-*values were determined from one-way ANOVA and post hoc Tukey’s test; *n.s*.: not significant. **c** Representative confocal and Imaris 3D images for astroglial localization of Spinal motor neuron-derived CD63-GFP^+^ exosomes and Cy5-miR-124-3p following sciatic nerve injection of AAV9-CaMKII-Cre and Cy5-miR-124-3p. Subpanels i: merge of tdT^+^ astrocyte with CD63-GFP and Cy5-miR-124; ii: merge of converted 3D tdT^+^ astrocyte with CD63-GFP and Cy5-miR-124; iii: merge of tdT and miR-124 signals on the upper part of the transected image in (ii) with 90 degree outward rotation; iv: merge of tdT and CD63-GFP signals on the upper part of the transected image in (ii) with 90 degree outward rotation; v–x: magnified view of (i) with either single or merged channels as indicated near the image; Scale bar: 20 μm (i), 10 μm (ii), 5 μm (iii–x); The dashed line indicates where the trans-section is. The upper part of the transected image turns 90 degree outward to show co-localized Cy5-miR-124-3p and CD63-GFP^+^ puncta (white arrow) inside tdT^+^ astrocytes. Yellow arrows: Cy5-miR-124-3p puncta; The magnified view was shown in v–x (white arrow: co-localized Cy5-miR-124-3p and CD63-GFP^+^ puncta; yellow arrow: Cy5-miR-124-3p; gray arrow: CD63-GFP^+^ puncta). The numbered puncta are the reference points to help orient the converted 3D image. Expression levels of miR-124-3p (**d**) and pri-miR-124 (**e**) in astrocytes isolated by FAC sorting from cortex of Bac *aldh1l1*-eGFP mice (P60-80) following GW4869 injections (daily i.p. for 28 days). *n* = 6 mice/group; U6 small nuclear (sn) RNA was used as the endogenous control. *P-*values were determined using two-tailed unpaired *t*-test; *n.s*. = not significant. The data was presented in the box and whisker plot with defined elements, median (center line), upper and lower quartiles (bounds of box), and highest and lowest values (whiskers)

We and others have previously found minimal miR-124-3p levels in cultured astrocytes^15,34^. Interestingly, eGFP^+^ astrocytes isolated by FAC sorting from primary neuron and astrocyte co-cultures have 12-fold higher miR-124-3p levels than that in astrocyte culture alone (Fig. 6a). Similarly, miR-124-3p levels in eGFP^+^ cortical astrocytes isolated from BAC *aldh1l1*-eGFP mice are developmentally upregulated from P8 to P40 (Fig. 6a). However, the transcription of endogenous miR-124-3p, indicated by the levels of its primary transcript pri-miR-124, in co-cultured or in vivo isolated astrocytes is not altered and remains as low as that in astrocytes alone (Fig. 6b). The significant increase of mature miR-124-3p but not pri-miR-124, in astrocytes from co-culture or in vivo conditions indicates that the increase of mature miR-124-3p levels in astrocytes is potentially a result of miR-124-3p transfer from neurons but not due to increased endogenous miR-124 transcription in these conditions. We also showed previously in vitro that direct addition of neuronal exosomes sufficiently increases miR-124-3p levels in astrocytes^15^. In addition, the Ct values for pri-miR-124 qPCR are oftentimes near background values (~ 32–34) in astrocytes in vitro or in vivo, confirming that endogenous miR-124 is indeed minimally transcribed in astrocytes.

To demonstrate that miR-124-3p can be transferred from neurons to astrocytes via exosomes in vivo, we took advantage of the retrograde transport property of sciatic nerves to SMNs and peripherally injected a mixture of AAV9-CaMKII-Cre virus and Cy5-miR-124-3p to sciatic nerves of *eaat2*-tdT^+^CD63-GFP^f/+^ double-positive mice to selectively deliver Cy5-miR-124-3p into SMNs and label SMN-derived exosomes. The injection paradigm is illustrated in Fig. 3f. Peripherally delivered Cy5-miR-124-3p is found inside SMNs (especially the SMN2 cell, Supplementary Fig. 4e) where the CD63-GFP is also induced. Interestingly, we also observed Cy5-miR-124-3p inside neighboring tdT^+^ astrocytes (Fig. 6c, i), either co-localizing with CD63-GFP (white arrow, Fig. 6c, v–x), an indication of exosome association, or by itself (yellow arrow, Fig. 6c, v–x). The intracellular localization of Cy5-miR-124-3p (alone or co-localized with CD63-GFP, yellow and white arrows) in the tdT^+^ astrocyte was further confirmed using the 3D astrocyte domain image (Fig. 6c, ii–iv). Additional examples of intracellular localization of Cy5-miR-124-3p (alone or co-localized with CD63-GFP, yellow and white arrows) and nearby CD63-GFP^+^ exosomes (gray arrows) in tdT^+^ astrocytes were shown in Supplementary Fig. 4f. How miR-associating exosomes are internalized and how quickly miRs are dissociated from exosomes in receipt cells is currently not well-understood. It is thus possible that Cy5-miR-124-3p quickly dissociates from CD63-GFP^+^ exosomes (not co-localized) inside astrocytes. It is also possible that the efficiency of retrograde transport of Cy5-miR-124-3p significantly influences how well its association with neuronal exosomes and transfer to astrocytes can be observed. Nevertheless, these in situ image analysis using our exosome reporter mice provided preliminary evidence that miR-124-3p can be associated with CD63-GFP^+^ exosomes and transferred into astrocytes. We further systemically inhibited exosome secretion by daily administration (i.p., 1.25 mg/kg) of GW4869, a neutral sphingomyelinase (nSMase) inhibitor known to block exosome secretion^35^ to BAC *aldh1l1*-eGFP mice. Although GW4869 inhibits exosome secretion from not only neurons but also other cell types, daily administration of GW4869 significantly decreases miR-124-3p levels in isolated eGFP^+^ astrocytes by 80% compared to sham controls (Fig. 6d) without changing pri-miR-124 levels in these astrocytes (Fig. 6e). GW4869-induced decrease of miR-124-3p in astrocytes, together with the increased miR-124-3p levels in astrocytes in vivo and in neuronal co-culture (Fig. 6a), further suggesting that miR-124-3p levels in astrocytes in vivo are mostly derived from (neuronal) exosomally transferred miR-124-3p, but not from its endogenous transcription in astrocytes.

### Inhibition of astroglial miR-124-3p reduces GLT1 expression

**Fig. 7:**
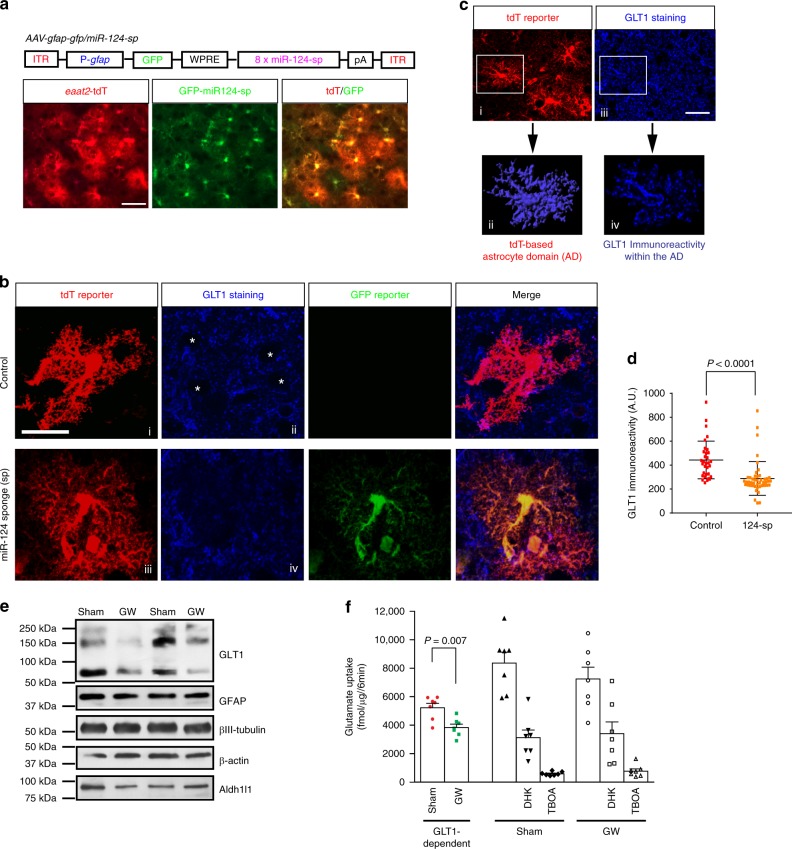
Selective inhibition of astroglial miR-124-3p and exosome secretion in vivo reduces functional GLT1 expression. **a** Diagram of AAV-*gfap*-GFP/miR-124-sponge (GFP-miR-124-sp) viral construct, and representative images of GFP-miR-124-sp expression in cortical tdT^+^ astrocytes of *eaat2*-tdT astrocyte reporter mice in vivo. Scale bar: 50 μm; **b** Representative images of cortical astrocytes from control and GFP-miR-124-sp injected *eeat2*-tdT astrocyte reporter mice. Subpanels i and iii: tdT^+^ astrocytes; ii and iv: GLT1 staining on astrocytes in (i) and (iii), respectively; Control: Adjacent non- transduced tdT^+^ astrocytes from AAV injected mice. White asterisks: neuronal somas. Scale bar: 10 μm; **c** Representative confocal and 3D images of cortical tdT^+^ astrocytes with GLT1 immunostaining. The astrocyte 3D domain (AD) was generated using Imaris software and then overlapped with GLT1 immunoreactivity. Subpanels i: tdT^+^ astrocytes; ii: magnified tdT^+^ astrocyte in (i) after 3D conversion; iii: GLT1 immunostaining; iv: GLT1 immunostaining signals inside the astrocyte domain in (ii); Only GLT1 immunoreactivity within the AD was quantified. Scale bar: 40 μm; **d** Quantification of GLT1 immunoreactivity within astrocyte domains after miR-124 sponge expression. *n* = 35–45 cells/group/4 mice; *P-*value was determined using two-tailed unpaired *t*-test; **e** Representative GLT1 immunoblot from the cortex of sham- and GW4869-injected mice (daily i.p., from P7 to P35). **f** Glutamate uptake levels from mouse cortex following sham or GW4869 injections. GLT1-dependent glutamate uptake was calculated by subtracting the remaining glutamate uptake with DHK treatment from total glutamate uptake. *n* = 7 mice/group; *P*-values were determined using two-tailed unpaired *t*-test. Error bars were presented in S.E.M

It is well established that astroglial GLT1 expression is dependent on neuronal stimulation^7^. Although we previously showed that neuronal exosomal miR-124-3p is sufficient to increase GLT1 in cultured astrocytes^15^, whether miR-124-3p is necessary for neuron-dependent up-regulation of GLT1 remains to be investigated. We therefore pre-transfected miR-124 antisense (miR-124-A/S) into astrocytes before adding neurons on the top of astrocytes. As shown in Supplementary Fig. 4b, c, pre-transfection of miR-124-A/S effectively abolishes 70% of neuron-induced GLT1 expression in neuron/astrocyte co-cultures. Pre-transfection of miR-124-A/S in astrocytes also significantly attenuates neuronal exosome-induced up-regulation of GLT1 expression^15^. The miR-124-A/S alone has no obvious effect on GLT1 expression in astrocyte culture alone (Supplementary Fig. 4d). In addition, we previously showed that in vivo injection of miR-124-A/S significantly decreases GLT1 protein expression and drastically abolishes glutamate uptake^15^. Given the essential roles of miR-124-3p in maintaining neuronal identity^33^, inhibition of overall (including neurons) miR-124-3p by miR-124-A/S likely induces neuronal damage and indirectly affects GLT1 expression. Indeed, severe neuronal dysfunction and abnormal axon growth has been observed when miR-124-3p is diminished in neurons^36^. Additionally, direct and specific inhibition of neuronal exosomal miR is not currently feasible. We therefore decided to selectively inhibit miR-124-3p expression only in astrocytes to determine whether miR-124-3p in astrocytes, presumably derived from neuronal exosomes, indeed regulates GLT1 expression in vivo. We employed the sponge (sp) strategy, in which complementary miR-124-3p sequences are expressed to selectively inhibit miR-124-3p. Based on the previously reported miR-124-3p sponge sequence^37^, we first generated the AAV5-*gfap*-GFP/miR-124-sponge (sp) construct and virus. In this construct, eight tandem miR-124-3p sponge sequences are inserted downstream of the *gfp* mRNA coding sequence and are subsequently produced when the *gfp* mRNA is transcribed (Fig. 7a). The effective inhibition of miR-124-3p by the miR-124-3p sponge has been previously demonstrated in both hippocampus and cortex in vivo^37^. AAV5-*gfap*-GFP/miR-124-sp virus was stereotaxically injected into the motor cortex of adult *eaat2*-tdT mice, in which the tdT reporter is selectively expressed in >80% of cortical astrocytes so that cortical astrocytes can be readily and reliably identified. AAV5-mediated expression of miR-124-sp is efficient and specific to cortical astrocytes, as evidenced by the widespread and selective co-localization of GFP fluorescence with tdT^+^ astrocytes (Fig. 7a).

To examine whether the selective inhibition of miR-124-3p in astrocytes alters GLT1 protein levels, we performed GLT1 immunostaining on cortical sections of AAV5-miR-124-sp-injected *eaat2*-tdT mice. Interestingly, GLT1 immunoreactivity inside individual tdT^+^ astrocytes that express miR-124-sp, indicated by GFP fluorescence (Fig. 7b, iv), is much reduced compared to GLT1 immunoreactivity of control (tdT^+^GFP^−^) astrocytes without AAV5 transduction (Fig. 7b, ii). Although there are often neuronal somas (* in control of Fig. 7b) that are GLT1 immunoreactivity negative and are surrounded by individual astroglial domains, it is unlikely that the reduced GLT1 immunoreactivity in tdT^+^GFP^+^ astrocytes is due to adjacent GLT1^-^ neuronal somas after carefully examining the *Z*-stack confocal images. GLT1 protein is known to be abundantly localized on the plasma membrane of highly ramified astroglial processes^38^, making its quantification in individual astrocytes often difficult and ambiguous. We previously showed that the full astrocyte domain can be well illustrated with the tdT reporter from *eaat2*-tdT mice by converting confocal astrocyte images into 3D images in the Imaris software^15^. The generation of the 3D domain for individual astrocytes (Fig. 7c, i–ii) facilitates accurate quantification of GLT1 immunoreactivity (Fig. 7c, iii–iv) within a given astrocyte domain (Fig. 7c, ii–iv). Quantification of GLT1 immunoreactivity from individual AAV5-*gfap*-GFP/miR-124-sp transduced (tdT^+^GFP^+^) and control (tdT^+^GFP^−^) astrocytes showed that the expression of miR-124-sp substantially decreases GLT1 immunoreactivity by 40% (*P* < 0.0001) in individual astrocytes (Fig. 7d), providing appealing in vivo evidence that astroglial miR-124-3p is important for GLT1 protein expression.

We further administered GW4869 in vivo (i.p. daily, P7 to P35) to block exosome secretion and found that exosome blockade decreases GLT1 protein expression (Fig. 7e) and specifically reduces 30% of GLT1-dependent glutamate uptake (Fig. 7f). It is possible that GW4869-mediated inhibition of ceramide production may induce non-specific consequences other than inhibition of exosome secretion, as ceramide is an essential component of lipid membrane. However, there is currently no specific approach to selectively inhibit neuronal exosome secretion. Meanwhile, no obvious toxicity or neuronal death was found in GW-administered mice (data not shown). Given the high abundance of GLT1 in the CNS with multiple regulatory mechanisms and > 50% reduction of GLT1-dependent glutamate uptake is more commonly observed in pathological conditions^39^, we consider a 30% reduction of glutamate uptake by exosome blockade highly significant. These results, especially from selective inhibition of astroglial miR-124-3p demonstrated that miR-124-3p in astrocytes, likely transferred through exosomes from neurons, acts non-cell autonomously to regulate astroglial glutamate uptake function in vivo.

### Suppression of GLT1-inhibiting miRs by miR-124-3p in astrocytes

**Fig. 8:**
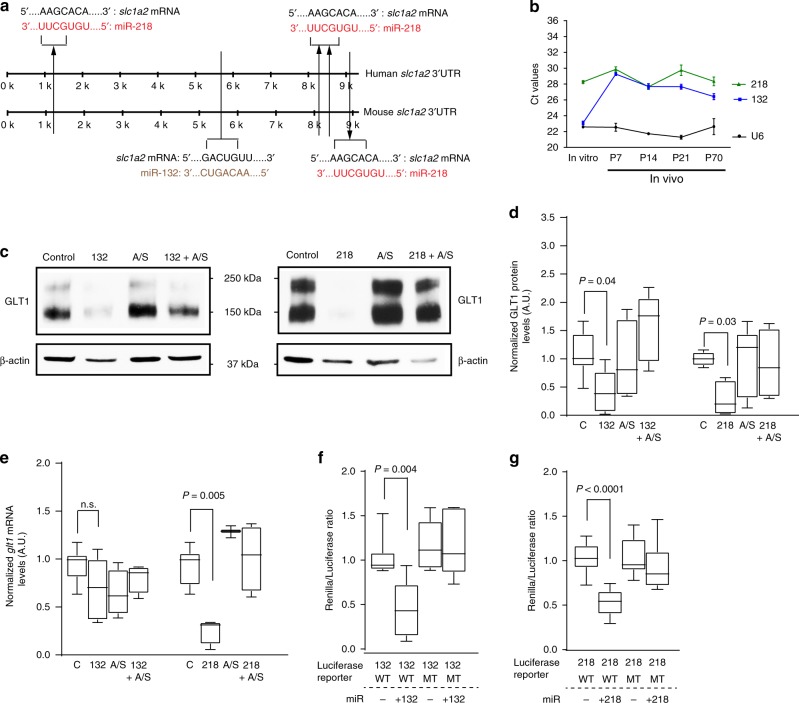
Identification of miRs that inhibit GLT1 protein expression in astrocytes. **a** Schematic diagram of the human and mouse *slc1a2* 3′ UTR with predicted binding sites for miR-218 and miR-132. TargetScanMouse was used for the *slc1a2* sequence analysis. Each arrow points to one binding site. **b** Expression levels of miR-218 and miR-132 in astrocytes in vitro (cultured astrocytes alone) and in vivo (at P7, P14, P21, and P70). Representative GLT1 immunoblots (**c**), quantification of GLT1 protein levels (**d**), and quantification by qPCR of *glt1* mRNA levels (**e**) from cultured primary astrocytes following transfection of miR-132 or miR-218 mimics, miR-132 or miR-218 antisense (A/S), and miR-132 or miR-218 along with their respective antisense. *n* = 6–8 independent experiments per condition. *P-*values were determined using one-way ANOVA and post hoc Tukey’s test. *n.s*.: not significant; **f** Wild-type (WT) and miR-132 mutant (MT) GLT1 3′ UTR luciferase activity in HEK 293 cells following miR-132 transfection. **g** Wild type (WT) and miR-218 mutant (MT) GLT1 3′ UTR luciferase activity in HEK 293 cells following miR-218 transfection. *n* = 3 independent experiments with six replicates per experiment per condition; one-way ANOVA and post hoc Tukey’s test; The data was presented in the box and whisker plot with defined elements, median (center line), upper and lower quartiles (bounds of box), and highest and lowest values (whiskers)

How miR-124-3p increases GLT1 protein levels in astrocytes is unknown. Although bioinformatic analysis of mouse and human *slc1a2* (that encodes GLT1) mRNA sequence revealed highly conserved miR-binding sites (Supplementary Fig. 5a) for multiple miRs, miR-124-3p has no conserved binding sites to the *slc1a2* mRNA. As miRs canonically inhibit protein expression by suppressing mRNA translation or disrupting mRNA stability^40^, we hypothesize that miR-124-3p upregulates GLT1 protein levels by suppressing GLT1-inhibiting molecules, such as miRs, in astrocytes. Indeed, several studies have begun to unveil GLT1-inhibiting miRs in astrocytes. To identify GLT1-inhibiting miRs in astrocytes, we therefore performed a screen using miR mimics in astrocyte cultures. The selection of miRs tested is based on their expression levels and number of *glt1* mRNA 3′ UTR-binding sites. Two miRs in particular, miR-132 and miR-218, have multiple predicted binding sites to the GLT1 3′ UTR (Fig. 8a) and are commonly detectable in both cultured and in vivo isolated astrocytes from Bac *aldh1l1*-eGFP mice (Fig. 8b). Transfection of miR-132 or miR-218 abolished 60% or 80% of GLT1 protein expression, respectively, in cultured astrocytes, which can be fully rescued by their specific antisense (Fig. 8c, d), while several other predicted *slc1a2* mRNA-binding miRs, miR-200c, -17, -30c, and -31 had no effect on GLT1 protein expression (Supplementary Fig. 5b, and data not shown). Transfected miR-218 but not miR-132 further specifically and drastically decreases *glt1* mRNA levels by 70% in cultured astrocytes (Fig. 8e). To determine whether miR-218 and miR-132 indeed bind to the *glt1* mRNA 3′UTR sequence at the predicted binding sites, we generated miR-132 and miR-218 wild-type (WT) and mutant (MT) *glt1* mRNA 3′ UTR luciferase reporter constructs and performed a luciferase assay in HEK 293 cells. Both miR-132 and miR-218 WT but not miR-132 and miR-218 MT *glt1* mRNA 3′ UTR luciferase constructs responded to co-transfected miR-132 or miR-218, respectively, with reduced luciferase reporter activity (Fig. 8f, g). These results suggest that both miRs are able to bind to the *glt1* mRNA 3′UTR sequence. However, the binding of miR-132 likely reduces GLT1 protein levels through suppression of *glt1* mRNA translation without affecting *glt1* mRNA levels, while miR-218 binding to the *glt1* mRNA disrupts its mRNA stability and subsequently decreases GLT1 protein expression.

**Fig. 9:**
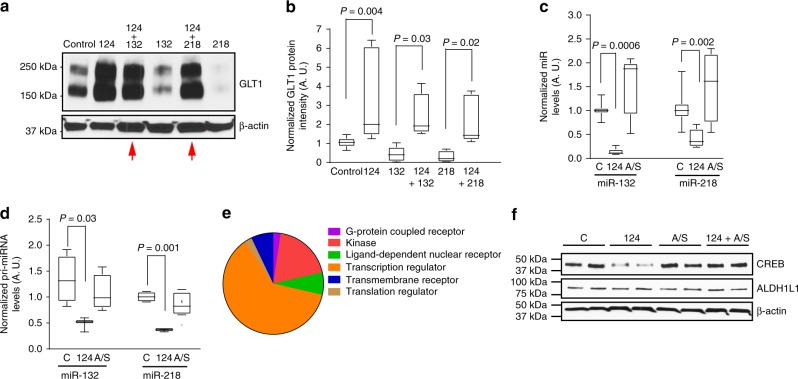
MiR-124-3p increases GLT1 expression by suppressing GLT1-inhibiting miRs in astrocytes. Representative GLT1 immunoblot (**a**) and quantification (**b**) following transfection of individual miRs (miR-124, miR-132, or miR-218) or co-transfection of miR-124-3p/miR-132 or miR-124-3p/miR-218 mimics in astrocyte cultures. *n* = 6–8 independent transfections per condition. *P-*values were determined from one-way ANOVA and post hoc Tukey’s test. Levels of miR-132 and miR-218 (**c**) and pri-miR-132 and pri-miR-218 (**d**) in astrocyte cultures following miR-124-3p transfection. *n* = 9 independent transfections per condition. β-actin mRNA was used as endogenous control for quantification of pri-miR-132. *P-*values were determined using one-way ANOVA and post hoc Tukey’s test. **e** Pie chart showing functional categories of genes that are expressed in astrocytes and are also predicted miR-124-binding targets. TargetScan was used to identify putative miR-124-3p target mRNAs. Ingenuity pathway analysis (Qiagen) was used for characterizing functional categories. Only genes that are expressed in both human and mouse astrocytes (FPKM > 10, mouse and human^42^ and have at least two predicted miR-124-3p-binding sites were included in generating the pie chart. **f** Representative Creb1 immunoblot from primary astrocytes following transfection with miR-124-3p and miR-124 antisense. The data was presented in the box and whisker plot with defined elements, median (center line), upper and lower quartiles (bounds of box), and highest and lowest values (whiskers)

To test whether miR-124-3p suppresses miR-132 and miR-218 in upregulating GLT1 expression in astrocytes, miR-124-3p was co-transfected with either miR-132 or miR-218 into cultured astrocytes and GLT1 protein expression was examined. Interestingly, miR-132- and miR-218-mediated inhibition of GLT1 expression was completely blocked by co-transfected miR-124-3p (red arrows, Fig. 9a), suggesting that miR-124-3p is able to strongly suppress miR-132 or miR-218’s inhibitory effects on GLT1 expression (quantification in Fig. 9b). Transfection of miR-124-3p further significantly decreases miR-132 and miR-218 (Fig. 9c) but not other miR levels, such as miR-361 and miR-143 (Supplementary Fig. 5c, d), in cultured astrocytes. Importantly, transfection of miR-124-3p also significantly reduces expression levels of the endogenously transcribed primary transcripts of miR-132 (pri-miR-132) and miR-218 (pri-miR-218), respectively (Fig. 9d), suggesting that miR-124-3p is able to suppress the endogenous transcription of miR-132 and miR-218 in astrocytes. We also found near background pri-miR-132 and pri-miR-218 levels in neuronal exosomes, consistent with previous observations that no pri-miRNA is found in exosomes^22^.

While the transcriptional regulation of miR-218 is unknown, the transcriptional factor Creb1 has been shown to play a key activating role in miR-132 transcription^41^. Interestingly, many of predicted miR-124-3p targeting mRNAs encode transcriptional factors (including Creb1) and other functional proteins such as transporters, channels, etc. (Fig. 9e) and are also expressed in both mouse and human adult astrocytes, based on TargetScan analysis and Ingenuity analysis of conserved mouse/human astrocyte RNA-seq datasets^42^. We then tested whether miR-124-3p indeed decreases Creb1 expression in astrocytes. As shown in Fig. 9f, miR-124-3p transfection decreases Creb1 expression by 80% in cultured astrocytes, which can be fully rescued by co-transfected miR-124-3p antisense (Fig. 9f), providing appealing evidence that miR-124-3p suppresses transcription of endogenous miR-132 by inhibiting Creb1 expression. Although miR-132 and miR-218 are also detected in neurons^43,44^, they are not enriched or selective in neuronal exosomes based on our miR microarray (data not shown). They also appear not to significantly transfer into astrocytes, as their levels in astrocytes co-cultured with neurons are only modestly increased (miR-132) or not changed (miR-218) when compared to their levels in astrocyte culture alone, far less than the significant miR-124 increase (Supplementary Fig. 5e). Thus, it is unlikely that transferred miR-132 plays a significant role in inhibiting GLT1 expression in astrocytes.

## Discussion

Current studies on exosome-mediated intercellular signaling are mostly limited to cultured models. Although previous detection of EVs/exosome markers from human cerebrospinal fluid (CSF) samples indicates that these vesicles are secreted in the CNS^45^, the in vivo localization of EV/exosomes, especially in the neuronal compartment, was unknown. By developing cell-type specific exosome reporter mice and performing subsequent confocal and immuno-EM analysis, we showed extracellularly localized CD63-GFP^+^ vesicles induced by neuronal CaMKII-Cre, providing the first in situ evidence that CD63^+^ vesicles can be secreted from neurons in the brain. Both immuno-EM analysis from brain sections and confocal analysis from cultured neurons found that neuronal Cre-induced intracellular CD63-GFP^+^ puncta are mostly localized in peri-nuclear soma and dendrites but not in axons of neurons. This is in contrast to observed multivesicular body (MVB) localization in pre-synaptic terminals at the NMJ in the fly model^18^. This may reflect the difference in experimental models (mouse vs. fly) or specific types of synapses (neuron to neuron vs. neuron to peripheral).

**Fig. 10:**
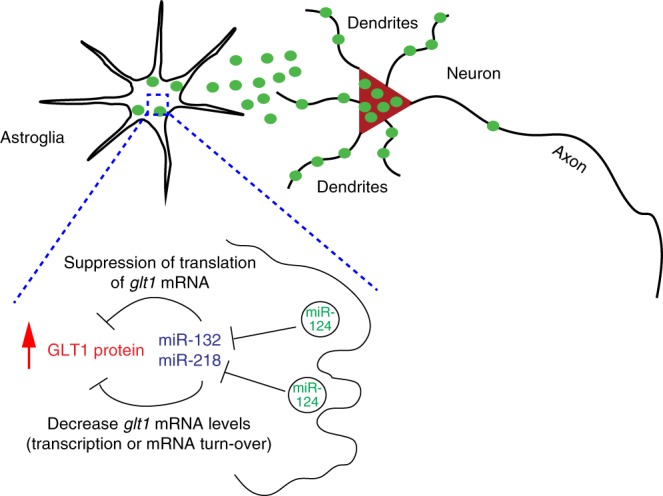
Schematic diagram of exosomal miR-124-3p in neuron to astroglial communication. Green solid circles: neuron-secreted exosomes; In this model, miR-124-3p-containing neuronal exosomes are released somatodendritically and can be internalized into astrocytes to increase GLT1 protein by genetically suppressing downstream miRs

Previous studies have suggested that exosome secretion is regulated by neuronal activity^17,46^. Our observation of the predominant somatodendritic localization of neuronal CD63-GFP^+^ puncta, however, points to an alternative mechanism for neuronal secretion of exosomes, i.e., neuronal activity-dependent release of exosomes from post-synaptic soma or dendrites (Fig. 10). Indeed, retrograde transfer of post-synaptically released vesicular signals has been observed and is implicated in synaptic functions^47^. Secretion of EVs/exosomes has also been found from non-neuron CNS cell types. We showed that CD63-GFP^+^ ILVs can be induced in all other CNS cell types^48^, thus our generated CD63^f/f^ exosome reporter mice also allow the examination of the localization of exosomes from other CNS cell types. In addition, EVs/exosomes have been associated with amyloid β and tau in Alzheimer’s disease (AD)^49,50^, α synuclein in Parkinson’s disease (PD)^51^, and TDP43, SOD1, and C9ORF72-derived dipeptide repeat (DPR) in amyotrophic lateral sclerosis (ALS)^52,53^, and other pathological conditions. It has been hypothesized that exosome signaling may facilitate spreading of pathogenic protein aggregates in neurodegenerative conditions^20^. Therefore, our mouse tool will allow characterization of exosome dynamics, especially in a cell-type-specific fashion, in neurodegenerative diseases, facilitating our understanding of the in vivo role of cell-type-specific exosome signaling in these pathological conditions. As the induced expression levels of CD63-GFP can be affected by induction conditions (dose of 4-OHT, titer of AAV-Cre virus, and induction period, etc.), it is beneficial to optimize the induction condition to avoid excessive overexpression of exogenous protein (CD63-GFP) and minimize potential-associated adverse effects.

Our current study presented exploratory in vivo evidence to support a previously unrecognized mode of communication from neurons to glia in the CNS. In this communication, neuron-specific miRs can be potentially transferred into astrocytes, likely from the post-synaptic somatodendritic compartment, through secreted exosomes (Fig. 10), which is in stark contrast to the typical pre-synaptically released neurotransmitter-mediated activation of receptors on astroglial surface. This is likely not only happening from neurons to astrocytes, but also to other glial cells. Indeed, miR-124-3p levels were not detected in cultured microglia^34^ but were abundant in microglia isolated from in vivo^54^, similar to miR-124-3p expression observed in astrocytes. In terms of downstream mechanisms, exosomally transferred miRs (miR-124-3p) directly alter genetic regulation in astrocytes by suppressing downstream miRs to regulate astrocyte (glutamate uptake) functions. This is also distinct from the previously characterized gliotransmission as the downstream mechanism following activation of astroglial surface receptors. As miRs are typically acting on mRNAs cell autonomously, it is a very intriguing observation that an exogenous miR (miR-124-3p) acts non-cell autonomously and regulates endogenous miR expression in astrocytes (Fig. 10).

Although we focused on identifying GLT1-binding miRs as downstream targets of miR-124-3p to increase GLT1 in astrocytes, it is likely that miR-124-3p also acts on other factors (including transcriptional factors) to regulate GLT1 expression, which is being investigated in a separate study. Notably, there are a number of astrocyte expressing genes encoding various functional protein categories (transcriptional factors, transporters, receptors, and channels) that are also predicted to be miR-124-3p’s target mRNAs (Fig. 9e). We showed that miR-124-3p decreases one of its predicted target mRNA encoding the transcriptional factor Creb1. By affecting the translation of these target mRNAs, neuronal exosomally transferred miR-124-3p is potentially able to modulate additional pathways/functions in astrocytes. Moreover, we identified a number of additional highly expressed or selective/enriched miRs other than miR-124-3p in neuronal exosomes, which could serve as additional neuronal signals to be transferred into astrocytes to modulate astrocyte functions, especially the miRs that are not detected in cultured astrocyte alone. In addition to the miRs, other categories of cargo (mRNA and protein) have begun to be characterized in neuronal exosomes^16,55^, though *glt1* mRNA and GLT1 protein was not detected in neuronal exosomes (data not shown). Whether these cargoes are transferred into astrocytes remains to be investigated.

In summary, our current study provided appealing evidence for an exosomal miR-mediated neuron to glial signaling, potentially opens a new frontier in understanding intercellular communication in the CNS. Future mechanistic studies of exosome-mediated neuron to glia signaling will provide important insights about how this signaling pathway contributes to CNS physiology and pathology.

## Methods

### Animals

The CD63-GFP^f/f^ knock-in mice were generated by homologous recombination. The human CD63-copGFP-6xHis cassette was subcloned onto the Rosa-CAG targeting vector, downstream of the CAG promoter and upstream of the 3′ arm, to generate the final CD63-copGFP-6xHis targeting vector. The copGFP is a variant of GFP, cloned from copepod *Pontellina plumata*. The copGFP has very bright fluorescence and is well resistant to photobleaching with no apparent cell toxicity^56^. The targeting vectors were linearized and transfected into the Embryonic Stem (ES) cell line to induce homolog recombination. G418-resistant ES clones were screened by Southern blot analysis and were subsequently injected into C57BL/6J blastocysts to obtain chimeric mice following standard procedures. Both ES cell transfections and blastocyst injections were performed by Biocytogen (Worcester, MA). Chimeric mice were bred with C57BL/6J mice to obtain germline transmission. CaMKII-Cre^ERT^ transgenic, (#012362) and WT mice (C57 BL6 background) were obtained from The Jackson Laboratory. The *eaat2*-tdT transgenic mice were previously generated^57^, Bac *aldh1l1*-eGFP mice were obtained from the GENSAT project through The Jackson Laboratory. CD63-GFP^f/f^ and CaMKII-Cre^ERT^ mice were bred to generate CaMKII-Cre^ERT^CD63-GFP^f/+^ mice. Both male and female mice were used in all experiments. All mice were maintained on a 12 h light/dark cycle with food and water ad libitum. Care and treatment of animals in all procedures strictly followed the NIH Guide for the Care and Use of Laboratory Animals and the Guidelines for the Use of Animals in Neuroscience Research. Animal protocols used in this study has been approved byTufts University IACUC committee.

### Drug administration

4-hydroxytamoxifen (4-OHT) (Sigma) was resuspended at 20 mg/mL in ethanol and diluted in sunflower seed oil at a final concentration of 2 mg/mL in 10% of ethanol. 4-OHT was given through i.p. injections (i.p.). The GW4869 compound (1.25 mg/kg, Sigma) was dissolved in Dimethyl Sulfoxide (12.5 mg/ml) and was also administered i.p. (daily) for 28 days from P7 to P35.

### Primary neuronal and astrocyte culture and transfection

For cortical astrocyte cultures, P0-P3 mouse pups were decapitated, and cerebral cortices were removed and transferred into 37 °C culture medium (Dulbecco Minimum Essential Medium, DMEM, supplemented with 10% FBS (fetal bovine serum) and 1% penicillin/streptomycin) for dissection. Meninges were stripped and cortices were minced and placed into 0.05% trypsin solution for 10 min in a 37 °C water bath. The enzymatic reaction was stopped by addition of astrocyte culture medium. The tissue was washed twice with astrocyte medium and then gently dissociated by trituration with a fire-polished Pasteur pipette. Dissociated cells were filtered through a 70 μm strainer to collect a clear astrocyte cell suspension. For oligodendrocyte cultures, oligodendrocyte progenitor cells (OPCs) were separated through shaking overnight (250 rpm, 37 °C) at day 8 of glial cultures and recovered in DMEM with 10% serum for 4 h^58^. Subsequently, the medium was switched to DMEM/F12 supplemented with 2% B27, 1% FBS, 20 ng/mL PDGFRα and 10 μg/mL bFGF for 48 h and for another 5 days without PDGFRα and bFGF. For microglia cultures, primary microglia were also isolated with the ‘shaking off’’ method^59^ (150 rpm, 37 °C for 2 h) at day 10 of the glial cultures. Cultures were maintained in DMEM supplemented with 10% FBS. For neuronal primary cultures, cortical neuronal cells were isolated from embryonic day 14–16 mouse brains. The neuron culture medium was composed of neurobasal medium, 2% B27 neurobasal supplement, 2 mM glutamine, 1% of 100x GlutaMAX, and 1% penicillin–streptomycin. The brain dissociation procedure was similar to the astrocyte isolation procedure described above. Freshly prepared neurons were then plated on cell culture dishes (8 × 10^6^ cells). AAV8-CaMKII-Cre_mCherry (1 μL, 1 × 10^12^ particles/mL), AAV5-GFAP-Cre (1 μL, 1 × 10^12^ particles/mL), or AAV5-PGK-Cre (1 μL, 1 × 10^12^ particles/mL) was added into the primary neuronal, astrocyte, or oligodendrocyte/microglia cultures, respectively, to induce the expression of CD63-GFP.

Primary astrocyte transfection in a six-well plate with different miRs, miR-A/S (antisense), or a mixture of miRs and miR-A/S (all final conc. 25 nM) was performed with Dharma FECT Reagent (Thermo Fisher) following the manufacturer’s instructions. For HEK 293 cell transfection, HEK 293 cells that were seeded 1 × 10^4^ per well in 96-well plates the day prior to transfection were incubated with 0.1 µg luciferase reporter construct alone, or luciferase reporter construct with individual miR mimics (100 nM, Thermo Scientific) using Dharma FECT Reagent. Mock transfected cells were transfected with the 3′UTR-free pmirGLO vector alone. Scramble small RNA was obtained from Dharmacon, Inc.

### Stereotaxic delivery of AAV and oligos in vivo

AAV8-CaMKII-Cre_mCherry (1 μL, 1 × 10^12^ particles/mL) or the mixture of AAV8-CaMKII-Cre (1 μL) with Cy5-miR-124 (5 μg/μL, 2 μL) was stereotaxically injected into the motor cortex (Bregma 1, *X* = 1.5 mm, *Z* = 0.75 mm) of CD63-GFP^f/+^ mice (P30-60). AAV8-CaMKII-Cre_mCherry, AAV5-*gfap*-Cre, and AAV5-*pgk*-Cre viruses were obtained from the University of North Carolina Vector Core (Chapel Hill, NC). AAV-*gfap*-GFP/miR-124-sponge construct was generated by replacing the *synapsin* promoter in plasmid AAV-*synapsin*-GFP/miR-124-sponge (a gift from Dr. Johan Hakobsson, Lund University, Sweden) with the *gfap* promoter from plasmid pGfa2-nLac (Addgene, #53126). Primers used for cloning (F: 5′- tatatagggcccgcggccgcacgcgtgatctaa-3′; R: 5′- tatataaccggtggtggcgtcgactctagaccccg-3′). AAV5-*gfap*-gfp/miR-124-sponge virus was generated by the Boston Children’s Hospital Viral core (Boston, MA). Mice were anesthetized with a cocktail of Ketamine/Xylazine (ketamine 95 mg/kg, and xylazine 10 mg/kg). Post-operative care included injections of buprenorphine according to the IACUC requirement. Animals were perfused 14 days after injections.

### Immuno-EM microscopy

Immuno-EM was carried out in the Harvard EM facility. Adult mice (P60) were perfused with 4% paraformaldehyde (PFA) and 0.1% glutaraldehyde. The brain was dissected out and post-fixed in 4% PFA for 2 h and then brain slices (100 μm) were prepared using vibratome. The slices were then quenched, permeabilized, and blocked in blocking buffer (3% bovine serum albumin, 5% normal donkey serum, and 0.1% Triton-X-100) at 4 °C. Anti-human CD63 (BD Pharmingen, #556019), anti-mouse CD63 (MBL, # D263-3) or GFP (Abcam, #6556) antibody was then added and incubated overnight at 4 °C. After wash, slices were incubated with Protein A-gold for 1 h at 25 °C. The images were taken using the JEOL 1200EX transmission electron microscope.

### Crude synaptosome preparation and glutamate uptake assay

Glutamate uptake assay was performed with crude synaptosome preparation from mouse cortices using the 0.32 M sucrose centrifugation method. After total protein determination by Bradford assay (Bio-Rad), 1 μCi L-^3^H glutamate and 100 μM non-labeled glutamate were mixed with Na^+^ uptake buffer (in mM; Tris 5, HEPES 10, NaCl 140, KCl 2.5, CaCl_2_ 1.2, MgCl_2_ 1.2, K_2_HPO_4_ 1.2, Glucose 10, total volume 275 μL) then added into 25 μL of each synaptosome sample in 96-well multiscreen High-throughput screening (HTS) filter plates (Millipore). After 6 min incubation, uptake was terminated by putting samples into an ice bath. Samples were then filtered using the Steriflip vacuum filtration system (Millipore) and washed 6x with ice-cold Phosphate-buffered saline (PBS) while continually filtering the samples. Each filtered 96-well membrane was excised out and transferred into vials for scintillation counting. Dihydrokainate (DHK, 500 μM) or DL-*threo*-β-Benzyloxyaspartic acid (DL-TBOA, 500 μM) was added into appropriate wells in glutamate uptake assay. Disintegration per min (DPM) value was normalized by total protein concentration and converted to fmol/μg/min unit. GLT1-dependent glutamate uptake was calculated by subtracting remaining glutamate uptake activity with DHK treatment from the total glutamate uptake activity.

### Immunoblot

Anti-mouse CD63 (MBL, # D263-3), anti-human CD63 (MBL, # MEX002-3), anti-CD81(1:200, clone B-11, Santa Cruz), anti-Tsg101 (1:100, clone C-2, Santa Cruz), anti-His-tag (1:100, clone AD1.1.10, Santa Cruz), anti-GFP (1:100, clone N86/8, NeuroMab), anti-EEA1 (1:100, clone G-4, Santa Cruz), anti-Rab7 (1:1000, clone D95F2, Cell Signaling Technology), anti-Calregulin (1:200, clone F-4, Santa Cruz), anti-GM130 (1:100, clone NN2C10/1, Santa Cruz), anti-Histone H2A.Z (1:400, 2718, Cell Signaling Technology), anti-β-actin (1:1000, A1978, Sigma), anti-GFAP (1:5000, Z0334, Dako), anti-β-III tubulin (1:1000, MAB1195, R&D system), anti-Creb1 (1:2000, 06-863, Sigma), and anti-Aldh1l1 (1:20, 73-140, Neuromab) were used. Mouse cerebral cortices, neuronal pellets, and exosome fractions were homogenized with lysis buffer (Tris-HCL pH 7.4, 20 mM, NaCl 140 mM, EDTA 1 mM, SDS 0.1%, Triton-X 1%, Glycerol 10%). Protein inhibitor cocktail (P8340, Sigma) was added in a 1/100 dilution to this lysis buffer prior to tissue homogenization. Total protein amount was determined by Bradford protein assay. Ten micrograms of cortical lysate or 20 μg of neuronal/exosomal lysate were loaded on 4–15% gradient sodium dodecyl sulfate polyacrylamide gel electrophoresis gels. Separated proteins were transferred onto a Polyvinylidene difluoride (PVDF) membrane (Bio-Rad) for 1 h. The membrane was blocked with 3% Bovine serum albumin (BSA) in TBST (Tris buffer saline with 0.1% Tween 20) then incubated with appropriate primary antibody overnight at 4 °C. On the following day, the membrane was exposed to HRP-conjugated goat anti-rabbit secondary antibody (1:5000) diluted in TBST. Bands were visualized on CL-XPosureTM film (Thermo Scientific) or Chemidoc MP imaging system (Bio-Rad) by ECL Plus chemiluminescent substrate (Thermo scientific). Standard Coomassie blue staining procedures were used. Exposure time was optimized for detecting different proteins.

### Sciatic nerve injection of AAV9-CamKII-Cre

The *eaat2*-tdT^+^CD63-GFP^f/+^ (P30-45) mice were deeply anaesthetized for surgery with ketamine (100 mg/kg) plus xylazine (10 mg/kg) in saline by i.p. injection. The hindquarter of the nerve to be injected was shaved using electric clippers, and the skin was sterilized using betadine and sterile alcohol. A horizontal incision through the skin was made on the mouse hind limb. The sciatic nerve was located by opening the fascial plane between the gluteus maximus and the anterior head of the biceps femoris. The sciatic nerve was then lifted from the muscle bellies and propped up using a spatula, taking care not to damage the nerve. A mixture of AAV9-CaMKII-Cre virus (2 μL, 1 × 10^13^ particles/mL from Penn Vector Core) and FluoroGold (1 μL) or a mixture of AAV9-CaMKII-Cre virus (2 μL, 1 × 10^13^ particles/mL from Penn Vector Core) and Cy5-miR-124-3p (2 μL, 100 μM) was then injected into sciatic nerves.

### Immunohistochemistry and confocal image acquisition and analysis

Animals were deeply anesthetized with Ketamine (100 mg/kg) + Xylazine (10 mg/kg) in saline by intraperitoneal (IP) injection and perfused intracardially with 4% paraformaldehyde (PFA) in PBS. The brains were dissected and kept in 4% PFA overnight at 4 °C, then cryoprotected by immersion in 30% sucrose for 48 h. Brains were embedded and frozen in OCT-Compound Tissu-Tek® (Sakura, Tokyo Japan). Coronal sections (20 µm) were prepared with a cryostat (Leica HM525) and mounted on glass SuperFrost + slides (Fisher Scientific). Slides were rinsed three times in PBS, then treated with blocking buffer (1% BSA, 5% goat-serum, and 0.2% Triton-X-100 in PBS) for 30 min at room temperature. Primary antibodies for GFAP (IF03L, Calbiochem), Map2 (clone AP20, Santa Cruz), NeuN (MAB377, Millipore), βIII-tubulin (MAB1195, R&D system), Olig2 (AB9610, Millipore), MBP (F-6 clone, Santa Cruz), GLT1 (a gift from Dr. Jeffrey Rothstein, Johns Hopkins University), Rab7 (clone D95F2, Cell Signaling Technology), and CD63 (MAB5417, R&D system) were incubated overnight at 4 °C in blocking buffer. After washing slides three times in PBS, corresponding secondary antibody (1:1000, Jackson ImmunoResearch, West Grove, PA) was added for 120 min at room temperature. The sections were rinsed three times in PBS before mounting. For fluorescent reporter mouse sections, reporter signals were not amplified by antibody staining. Low magnification images were taken using the Zeiss Axio imager with ApoTome except for the representative whole brain and spinal cord images, which were taken at x10 magnification using the Keyence microscope. Confocal images were taken using the Nikon A1R or Leica SPE confocal laser scanning microscope (15–20 μm *Z*-stack with 0.5 μm step) magnified with ×40(numerical aperture 0.8) or ×63 (numerical aperture 1.0) objectives.

For quantifying GLT1 immunoreactivity within individual astrocytes, astroglial domains from the somatosensory-motor cortex (layers 2–5) were used for analysis. Three-dimensional (3D) reconstruction of astroglial domains were first performed using Imaris (Bitplane), as we previously described^6^, using original confocal *Z*-stack images (40 × ) in Imaris software (Bitplane). For the quantification of CD63-GFP intensity from neuronal compartments of primary neuronal cultures, GFP intensity of the confocal images (63 × ) was first thresholded and then its intensity in each compartment of neurons was measured using the ROI function in Image J. The total axons, both proximal and distal, were included in the quantification. For the quantification of overall CD63-GFP^+^ puncta size, the green channel of the confocal image (taken at 63 × ) was thresholded to remove the background signals. The puncta were then analyzed using the particle analysis tool in Image J to obtain particle count and particle area from which the radius and diameter (μm) was calculated and calibrated based on the scale bar. For the quantification of the co-localization between GFAP immunoreactivity and CD63-GFP^+^ puncta, GFP and GFAP channels of confocal images (63 × ) were first thresholded to remove background and then merged to identify their co-localization using the co-localization plugin in Image J. The GFP channel image was also analyzed using the particle analysis function in Image J. To quantify the size and quantity of co-localized CD63-GFP puncta, co-localized signals were then manually aligned with CD63-GFP channel particle analysis to point to original CD63-GFP puncta that were overlapped with GFAP signals. For the quantification of co-localized CD63-GFP^+^/Cy5-miR-124 puncta inside GFAP^+^ astrocytes, co-localization between CD63-GFP^+^/Cy5-miR-124 was first performed, then their co-localization with GFAP was similarly analyzed and the triple co-localized puncta were quantified. All analysis was done randomly and blindly.

### Exosome purification and qNano particle analysis

Exosomes were prepared from neuron conditioned medium (NCM) from neuronal primary culture (8 × 10^6^ cells/10 cm dish) as previously described^15^. NCM was first spun at 300 x *g* for 10 min to remove cell debris, 2000  x *g* for 10 min at 4 °C, and then underwent a 10,000 x *g* centrifugation step for 60 min. The supernatant was passed through a 0.22 μm polyethersulfone (PES) filter (Merck Millipore, MA, USA). Finally, exosomes were pelleted by ultracentrifugation at 100,000 x *g* for 60 min at 4 ° C. Tunable resistive pulse sensing (TRPS) by qNano instrument^60^ (Izon Science, MA, USA) was used to measure the size distribution and quantity of particles in isolated exosome fractions, as described in the instrument. An aliquot of exosomes fraction or calibration particles included in the reagent kit were placed in the Nanopore (NP150, Izon Science). Samples were measured at 44.9 mm stretch with a voltage of 0.76 V at 1-pressure levels of 14 mbar. Particles were detected in short pulses of the current (blockades). The calibration particles were measured directly after the experimental sample under identical conditions. The data was processed using the Izon software (version 3.2).

### RNA isolation and miR/pri-miR qPCR, and miR synthesis

Total RNA was extracted from exosome fractions, neuronal cell pellets, or FAC-sorted cells using TRIzol reagent by following the manufacturer’s instructions. The quantity and quality of isolated RNA was determined using the Agilent BioAnalyzer with the nano or pico chip according to the manufacturer’s instructions. The miR was converted to cDNA using the TaqMan MicroRNA Reverse Transcription Kit (Applied Biosystems) with specific primers for each individual miRs (included in each miR kit) and control U6 small nuclear (sn) RNA (Applied Biosystems). qPCR was performed with matching miR probes and TaqMan 2x Universal PCR Master Mix. For pri-miR-124, pri-miR-132, pri-miR-218, *actb* (β-actin), and *glt1*, random primers (provided in Reverse Transcription Kit) were used for the reverse transcription. The relative quantity of pri-miR-124, pri-miR-132, pri-miR-218, beta actin, and *glt1* mRNA was measured by qRT-PCR using SyberGreen. Specific probes for each pri-miRNA were included in pri-miRNA TaqMan kit, *actb*: 5′ ggctgtattcccctccatcg 3′, 5′ ccagttggtaacaatgccatgt 3′; *glt1*: 5′ acaatatgcccagcaggtaga 3′, 5′ gacaccaaacacagtcagtga 3′. Individual miR double stranded mimics and antisense were synthesized (Thermo Scientific). Cy5-labeled miR-124-3p was synthesized (IDT DNA technologies) with a 2′ O-Methyl group on each nucleotide and a phosphoorothioate bond between the first and last three nucleotides.

### Affymetrix microRNA microarray

Neuronal and exosomal RNA was hybridized to GeneChip miRNA (Affymetrix, version 3.0 array) at Boston University microarray facility. Raw Affymetrix CEL files were normalized to produce probe set-level expression values for all mouse and control probe sets using Affymetrix Expression Console (version 1.3.0.187), using the robust multiarray average (RMA) and detection above background (DABG) commands. Analysis was limited to the 1111 known mouse microRNAs included on the array. Array quality was assessed by computing mean relative log expression (RLE) and percent Present calls in Expression Console. Analyses of variance were performed using the ‘f.pvalue’’ function in the SVA package (version 3.4.0). Pairwise differential expression of microRNAs was assessed with Student’s two-sample *t-*test performed on the coefficients of simple linear models created using the limma package (version 3.14.4). All microarray analyses were performed using the R program (version 2.15.1).

### Fluorescent-activated cell sorting of astrocytes

BAC *aldh1l1*-eGFP^+^ transgenic mice (P8, or P30-60) were used for FAC sorting at the Tufts FACS facility. Animals were deeply anesthetized with ketamine (100 mg/kg) + xylazine (10 mg/kg) in saline by i.p. injection and perfused intracardially with Hanks Buffered Salt Solution (HBSS) (Thermo Scientific). The brain was immediately dissected in cold Hanks buffer supplemented with glutamate receptor antagonists, 3 mM DNQX and 100 mM APV (Sigma, St. Louis, MO), and cut into small pieces. Cell suspension was prepared by following the manufacturer’s instructions in the neural tissue dissociation kit (Miltenyi Biotech, Auburn, CA). Briefly, small pieces of tissue were treated with papain enzymatic mix (37 °C, 15 min) and then digested with DNase I (37 °C, 10 min), followed by careful trituration. Cell mixtures were then filtered through a cell strainer (40–70 mm) and resuspended in cold HBSS solution (5–10 × 10^6^ cells/mL) for FACS. Cells were sorted by using MoFlo MLS high-speed cell sorter (Beckman Coulter) with Summit version 4.3 software. The whole procedure for cell suspension preparation and FAC sorting process was completed within 2–3 h. FAC-sorted cells were spun down and RNA was isolated from the cell pellet using standard TRIzol reagent.

### Generation of luciferase reporters and luciferase assay

The *glt1* mRNA 3′ UTR luciferase reporter construct containing three adjacent miR-218-binding sites was generated via PCR using mouse genomic DNA as a template using the following primers: 5′ tcatactcaaatcatgtcttctg 3′; 5′ ctttcactaagtgttttaactac 3′. The corresponding *glt1* 3′ UTR with miR-218 sites deleted was chemically synthesized (Genscript). The *glt1* 3′ UTR containing miR-132-binding sites was synthesized (Integrated DNA Technologies) as follows: Wild Type 5′ taacaacagactgttatccttatc 3′; Mutant 5′ taacaacatccttatc 3′. PCR products or synthesized oligos were cloned into the pmirGLO vector (Promega). Reporter constructs were confirmed by sequencing. Luciferase activity was measured 48 h after transfection of HEK 293 cells using the Dual-Glo luciferase kit (Promega). Coexpressed *Renilla* luciferase on the pmirGLO vector was used as an internal control to normalize the firefly luciferase activity.

### Brain slice preparation and neuronal dye-fill

Cortical brain slices were prepared from postnatal day 30–40 CamKII-CreER^+^CD63-GFP^f/+^ mice. Animals were anesthetized with a ketamine/xylazine cocktail (110 mg/kg/10 mg/kg); the brain was quickly removed and 300 μm cortical slices were cut using a vibratome (Leica VT1000, Leica Microsystems; Witzlar, Germany) in ice-cold artificial cerebrospinal fluid (aCSF) (in mM): KCl 3, NaCl 125, MgCl_2_ 1, NaHCO_3_ 26, NaH_2_PO_4_ 1.25, glucose 10, CaCl_2_ 2 and 400 μM l-ascorbic acid, with osmolarity at 300–305 mOsm, equilibrated with 95% O_2_-5% CO_2_. Slices were incubated at room temperature (RT) until needed. Whole-cell patch-current recordings from layer 2/3 or 5 somatosensory cortex pyramidal neurons were obtained by Axopatch 200B amplifier filtered at 2 kHz and sampled at 10 kHz with Digidata 1440 A (Molecular Devices, Sunnyvale, CA). Neurons were filled with Alexa-568 dye by applying negative pulse (1 Hz) until all neuronal processes were visualized (~ 15 min).

### Experimental design and statistical analysis

Sample size and statistical approach used for each experiment are described in each method section and in figure legends. Previous results and the G*Power (version 3.1) were used to estimate the reasonable sample size. All analyses were performed using GraphPad Prism 7. All values were plotted as individual values or box and whisker plot with all defined elements, including median (center line), upper and lower quartiles (bounds of box), and the highest and lowest values (wiskers). For graphs with error bars, error bars were presented in S.E.M. No custom code was used in the analysis. For multiple groups ( > 2), one-way analysis of variance (ANOVA) was used to analyze the variance, followed by a Tukey post-hoc test to compare multiple groups. For two-group comparison, two-tailed student t-test was used. Statistical significance was tested at a 95% (*P* < 0.05) confidence level and P values were shown in each graph.

### Reporting summary

Further information on research design is available in the Nature Research Reporting Summary linked to this article.

## Supplementary information

Supplementary Information

Reporting Summary

## Data Availability

No large datasets were generated from this study. All data supporting the findings of this study are available from the corresponding author on reasonable request.
