# Supplementary material for: Exosome reporter mice reveal the involvement of exosomes in mediating neuron to astroglia communication in the CNS
Source: Nat Commun. 2019 Sep 12;10:4136. doi: 10.1038/s41467-019-11534-w (PMC6742670; doi:10.1038/s41467-019-11534-w)
Supplement: Supplementary file 1 — Supplementary Information [file 41467_2019_11534_MOESM1_ESM.pdf]

Supplementary Information

Exosome reporter mice reveal the involvement of exosomes in mediating neuron to astroglia communication in the CNS

Men, et al.,

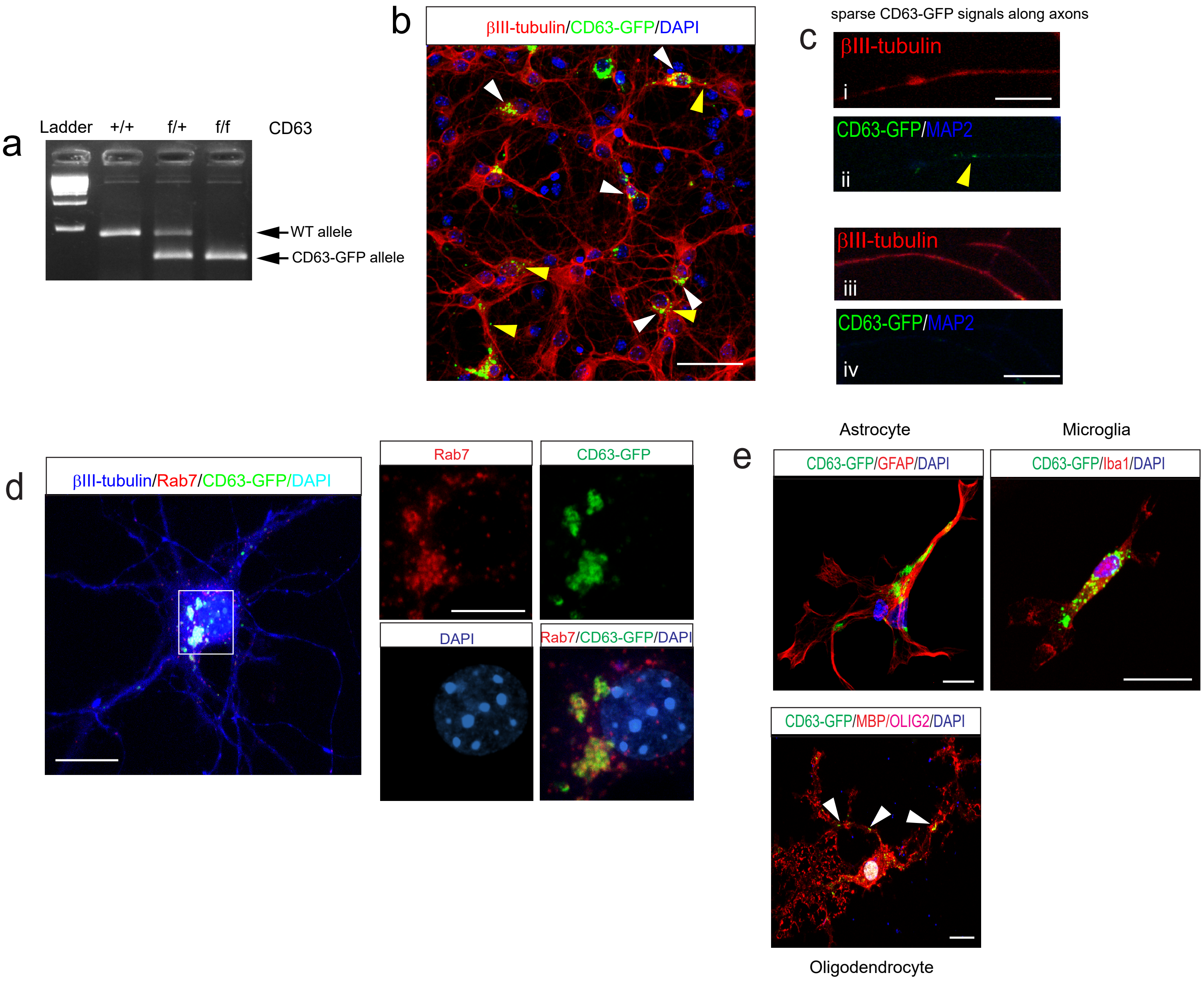

a
Ladder
+/+
f/+
f/f
CD63
WT allele
CD63-GFP allele
b
βIII-tubulin/CD63-GFP/DAPI
c
sparse CD63-GFP signals along axons
βIII-tubulin
i
CD63-GFP/MAP2
ii
βIII-tubulin
iii
CD63-GFP/MAP2
iv
d
βIII-tubulin/Rab7/CD63-GFP/DAPI
Rab7
CD63-GFP
DAPI
Rab7/CD63-GFP/DAPI
e
Astrocyte
Microglia
CD63-GFP/GFAP/DAPI
CD63-GFP/Iba1/DAPI
CD63-GFP/MBP/OLIG2/DAPI
Oligodendrocyte

Supplementary Figure 1: a, Representative PCR genotyping result of CD63-GFP$^{f/f}$ and CD63-GFP$^{f/+}$ mice; **b,** Representative image of CD63-GFP induction in cultured primary neurons by AAV8-CaMKII-Cre; Scale bar: 50μm; white arrows: CD63-GFP signals in soma; yellow arrows: CD63-GFP signals in neurites; **c,** Representative magnified axon images of primary neurons. Note very sparse CD63-GFP signals (yellow arrows) along axons (βIII-tubulin^{+}MAP2^{-}). Scale bar: 50 μm; **d,** Immunostaining of Rab7 in CD63-GFP^{+} primary neurons; Scale bar: 20μm (left) and 10μm (right); **e,** Representative images of CD63-GFP induction in astrocytes, microglia, and oligodendrocytes. Scale bar: 20μm. white arrows: intracellular CD63-GFP^{+} puncta.

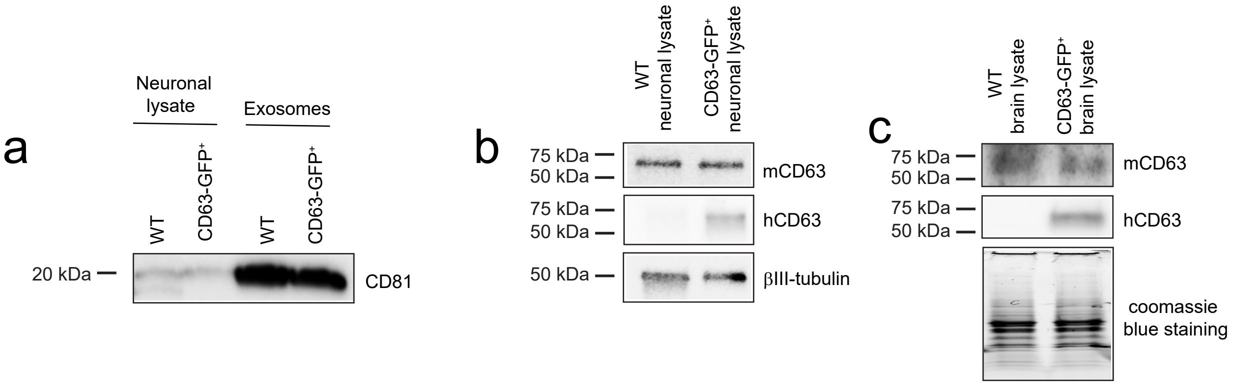

a
Neuronal lysate
Exosomes
WT
CD63-GFP+
WT
CD63-GFP+
20 kDa
CD81
b
WT neuronal lysate
CD63-GFP+ neuronal lysate
75 kDa
50 kDa
mCD63
75 kDa
50 kDa
hCD63
50 kDa
βIII-tubulin
c
WT brain lysate
CD63-GFP+ brain lysate
75 kDa
50 kDa
mCD63
75 kDa
50 kDa
hCD63
coomassie blue staining

Supplementary Figure 2: a, Representative immunoblot of exosomal marker CD81 in neuronal lysate and exosomes of both WT and AAV-CaMKII-Cre transduced CD63-GFP$^{f/+}$ primary neurons. **b,** Representative immunoblot of induced human CD63 and endogenous mouse CD63 from WT and CD63-GFP^{+} neuronal lysate. **c,** Representative immunoblot of induced human CD63 and endogenous mouse CD63 from WT and CaMKII-CreER^{+}CD63-GFP^{+} brain lysate. Coomassie blue staining was performed to show equal protein loading. Images were acquired using the Bio-Rad ChemiDoc imaging station.

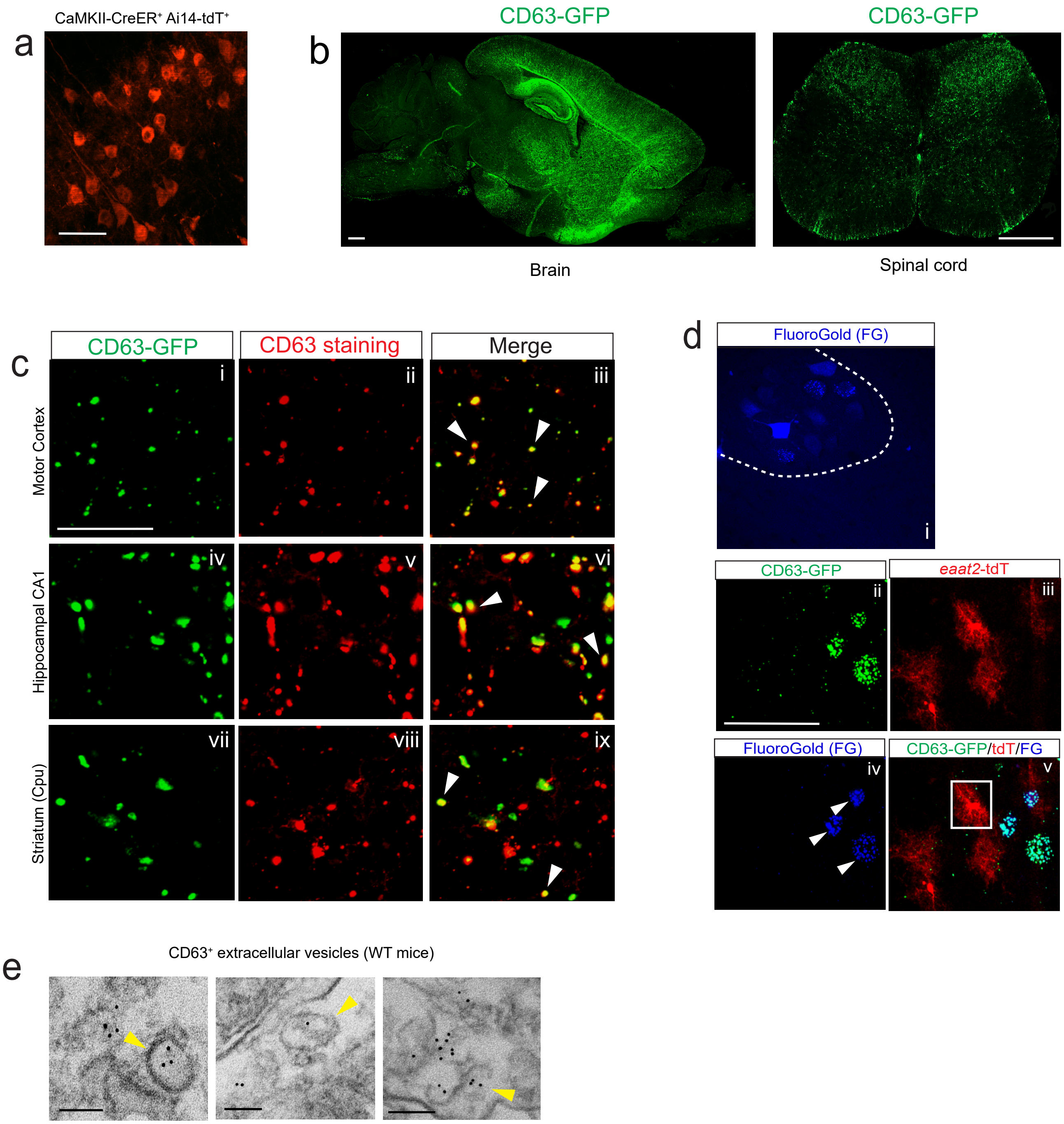

a
CaMKII-CreER+ Ai14-tdT+
b
CD63-GFP
CD63-GFP
Brain
Spinal cord
c
CD63-GFP
CD63 staining
Merge
Motor Cortex
Hippocampal CA1
Striatum (Cpu)
i
ii
iii
iv
v
vi
vii
viii
ix
d
FluoroGold (FG)
i
CD63-GFP
eaat2-tdT
ii
iii
FluoroGold (FG)
CD63-GFP/tdT/FG
iv
v
e
CD63+ extracellular vesicles (WT mice)

Supplementary Figure 3: a, A representative image of cortical tdT^+ neurons in CaMKII-$CreER^+$Ai14-tdT^+ mice. Scale bar: 50μm; Typical neuronal soma and neurites were labeled. **b,** CD63-GFP expression in the brain and spinal cord in CaMKII-$CreER^+$CD63-$GFP^{f/+}$ mice. A single dose of 4-OHT (10mg/kg) was given at P10. Mice were collected at P30. Scale bar: 1mm (brain) and 0.5mm (spinal cord); **c,** Immunostaining of endogenous mouse CD63 on motor cortex (iii), hippocampus (CA1, vi), and striatum (Cpu, ix), of CaMKII-$CreER^+$CD63-$GFP^{f/+}$ mice. Subpanels i, iv, and vii: CD63-GFP fluorescence; ii, v, and viii: CD63 immunostaining; iii, vi, and ix: merge of CD63-GFP fluorescence and CD63 immunostaining; White arrows: co-localization between CD63 immunostaining signals and induced CD63-GFP fluorescence; Scale bar: 25μm; **d,** Representative images of AAV9-CaMKII-Cre-induced CD63-GFP expression and FG labeling of spinal motor neurons in the lumbar cord of *eaat2*-tdT^+CD63-$GFP^{f/+}$ mice, dashed line to show the grey matter of lumbar cord (i); Scale bar: 100μm (ii-v); **e,** Representative $CD63^+$ extracellular vesicles from wild type brain sections (cortex and hippocampus). Yellow arrows: extracellularly localized $CD63^+$ vesicles; The anti-CD63 antibody specifically recognizing mouse CD63 was used. Scale bar: 100nm;

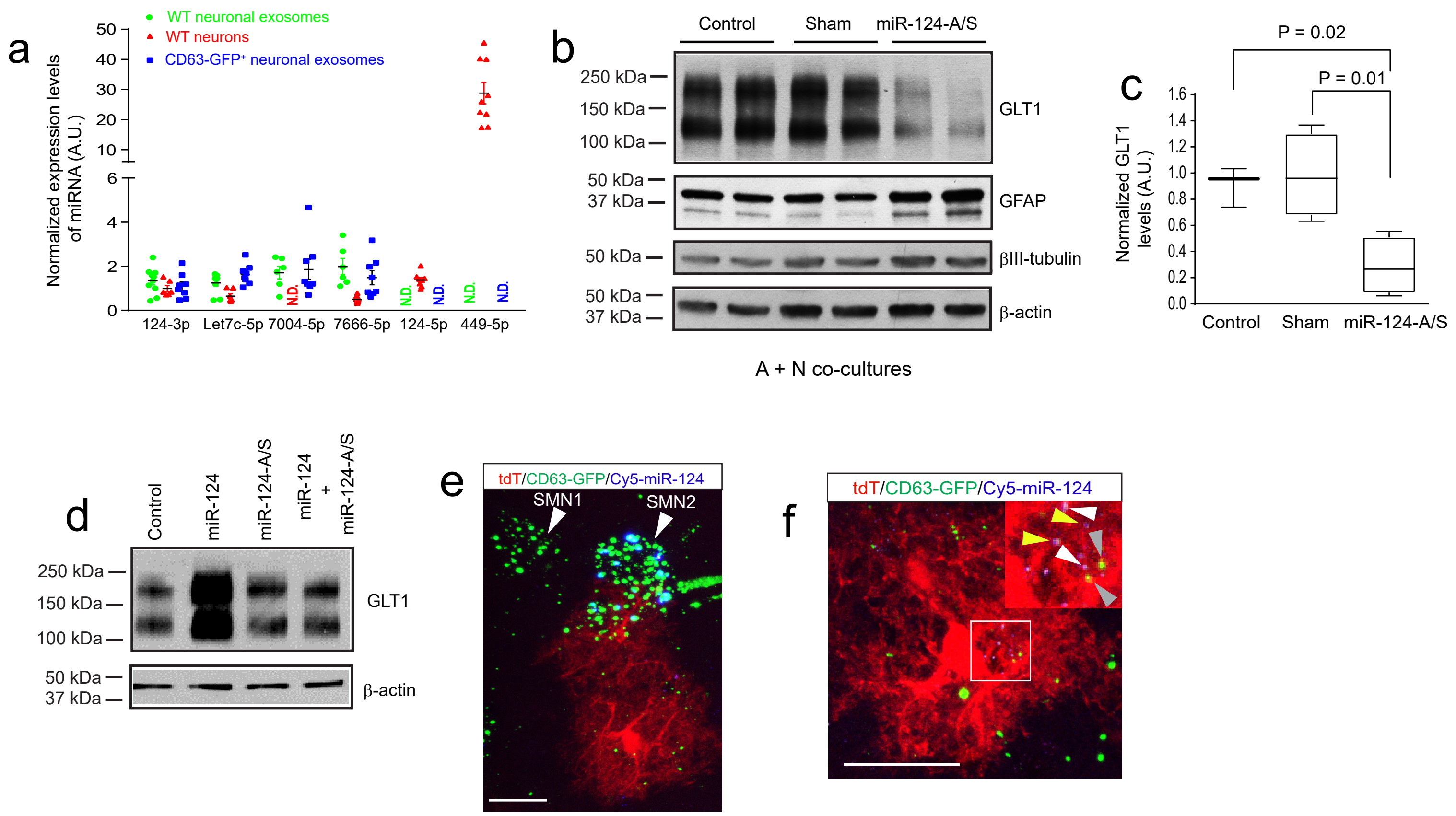

a
WT neuronal exosomes
WT neurons
CD63-GFP⁺ neuronal exosomes
Normalized expression levels of miRNA (A.U.)
124-3p
Let7c-5p
7004-5p
7666-5p
124-5p
449-5p
N.D.
b
Control
Sham
miR-124-A/S
250 kDa
150 kDa
100 kDa
GLT1
50 kDa
37 kDa
GFAP
βIII-tubulin
β-actin
A + N co-cultures
c
P = 0.02
P = 0.01
Normalized GLT1 levels (A.U.)
Control
Sham
miR-124-A/S
d
Control
miR-124
miR-124-A/S
miR-124 + miR-124-A/S
250 kDa
150 kDa
100 kDa
GLT1
50 kDa
37 kDa
β-actin
e
tdT/CD63-GFP/Cy5-miR-124
SMN1
SMN2
f
tdT/CD63-GFP/Cy5-miR-124

Supplementary Figure 4: a, Relative expression of representative miRs selective or enriched in neurons (WT) and neuronal exosomes (WT and CD63-GFP$^+$) by qPCR. All original Ct values are within 26-32 range, Ct > 34 is deemed not detected (N.D.). U6 small nuclear (sn) RNA was used as the endogenous control. n = 6-9 biologically independent samples per condition. Representative immunoblot of GLT1 (**b**) and quantification (**c**) in neuron and astrocyte (A + N) co-cultures following transfection of miR-124-A/S into cultured astrocytes; Sham: transfection of scramble small RNA (Dharmacon). n = 6/group from three independent experiments. P values were determined using One-way ANOVA followed by a Tukey post-hoc test. **d,** Representative immunoblot that miR-124-3p significantly up-regulates GLT1 protein expression in astrocyte cultures. e, Representative image of AAV9-CaMKII-Cre-induced CD63-GFP expression and delivery of Cy5-miR-124-3p into spinal motor neurons in the lumbar cord of *eaat2*-tdT$^+$CD63-GFP$^{f/+}$ mice. Scale bar: 20 μm; SMN1: a representative motor neuron with minimal retrograde transported Cy5-miR-124-3p; SMN2: a representative motor neuron with abundant retrograde transported Cy5-miR-124-3p; f, Representative image of the internalization of Cy5-miR-124-3p in the tdT$^+$ astrocyte. White arrow: co-localized Cy5-miR-124-3p with CD63-GFP$^+$ puncta; Yellow arrow: Cy5-miR-124-3p puncta; Gray arrow: CD63-GFP$^+$ puncta; Scale bar: 20 μm;

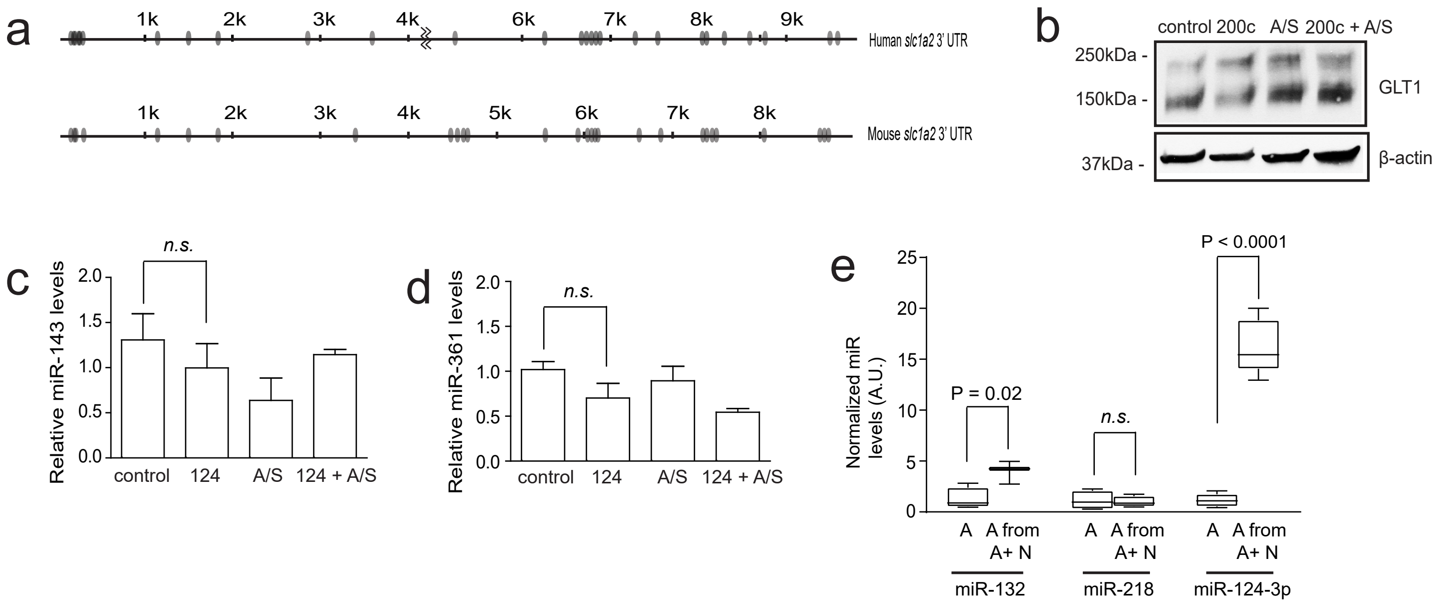
a
1k
2k
3k
4k
6k
7k
8k
9k
Human slc1a2 3' UTR
1k
2k
3k
4k
5k
6k
7k
8k
Mouse slc1a2 3' UTR
b
control
200c
A/S
200c + A/S
250kDa -
150kDa -
GLT1
37kDa -
β-actin
c
Relative miR-143 levels
n.s.
2.0
1.5
1.0
0.5
0.0
control
124
A/S
124 + A/S
d
Relative miR-361 levels
n.s.
2.0
1.5
1.0
0.5
0.0
control
124
A/S
124 + A/S
e
Normalized miR levels (A.U.)
25
20
15
10
5
0
P = 0.02
n.s.
P < 0.0001
A
A from A+ N
miR-132
miR-218
miR-124-3p

Supplementary Figure 5: a, Bioinformatic analysis of conserved miR binding sites on human and mouse *slc1a2* mRNA 3 UTR sequence. Grey oval circle: individual conserved miR binding site; **b,** GLT1 immunoblot from astrocyte cultures following miR-200c transfection. Expression levels of miR-143 (**c**) and miR-361 (**d**) following transfection of miR-124-3p or miR-124-A/S in astrocyte cultures. n = 4 independent experiments per condition. *n.s.*: not significant; **e,** Expression levels of individual miRs in cultured astrocytes alone (A) and astrocytes isolated from astrocyte and neuron co-cultures (A from A + N). n = 6 independent cultures per condition. P values were determined using one-way ANOVA followed by a Tukey post-hoc test.

Supplementary Table 1
miRs that are selectively epressed in neurons. N: neurons; E: exosomes; N.D.: not detected;

miR ID	FDR q	Expression in N (Log_2)	Expression in E
mmu-miR-23b-3p	1.56E-05	9.759672	N.D.
mmu-miR-30c-5p	2.5E-05	9.038566667	N.D.
mmu-miR-3535	1.56E-05	7.582142	N.D.
mmu-miR-3535	1.56E-05	7.582142	N.D.
mmu-miR-19b-3p	0.000133	8.650682667	N.D.
mmu-miR-18a-5p	1.85E-05	7.793825	N.D.
mmu-miR-151-5p	2.41E-05	8.876877	N.D.
mmu-miR-379-5p	4.48E-05	8.10003	N.D.
mmu-miR-344-3p	1.56E-05	7.274605333	N.D.
mmu-miR-677-3p	1.56E-05	7.016964	N.D.
mmu-miR-677-3p	1.56E-05	7.016964	N.D.
mmu-miR-30b-5p	1.56E-05	6.628535333	N.D.
mmu-miR-30b-5p	1.56E-05	6.628535333	N.D.
mmu-miR-30a-5p	2.8E-05	7.065342	N.D.
mmu-miR-137-5p	1.85E-05	7.061896333	N.D.
mmu-let-7f-5p	3.26E-05	7.835905333	N.D.
mmu-miR-139-5p	6.67E-05	7.743960333	N.D.
mmu-miR-434-3p	4.76E-05	7.837678	N.D.
mmu-miR-194-5p	2.41E-05	6.654628333	N.D.
mmu-miR-194-5p	2.41E-05	6.654628333	N.D.
mmu-miR-181c-3p	1.85E-05	6.600500667	N.D.
mmu-miR-181c-3p	1.85E-05	6.600500667	N.D.
mmu-miR-9-3p	7.39E-05	8.209668667	N.D.
mmu-miR-9-5p	0.00013	5.579854333	N.D.
mmu-miR-9-5p	0.00013	5.579854333	N.D.
mmu-miR-15a-5p	2.8E-05	6.064716667	N.D.
mmu-miR-181c-5p	1.85E-05	6.116027	N.D.
mmu-miR-181c-5p	1.85E-05	6.116027	N.D.
mmu-miR-331-3p	3.17E-05	6.67822	N.D.
mmu-miR-331-3p	3.17E-05	6.67822	N.D.
mmu-miR-7a-2-3p	2.41E-05	7.388512333	N.D.
mmu-miR-5121	1.85E-05	5.947028667	N.D.
mmu-miR-5121	1.85E-05	5.947028667	N.D.
mmu-miR-431-5p	3.17E-05	6.295634333	N.D.
mmu-miR-431-5p	3.17E-05	6.295634333	N.D.
mmu-miR-93-3p	2.41E-05	6.373812667	N.D.
mmu-miR-22-3p	2.93E-05	7.514736	N.D.
mmu-miR-362-5p	2.45E-05	5.691050667	N.D.
mmu-miR-362-5p	2.45E-05	5.691050667	N.D.
mmu-miR-137-3p	4.76E-05	5.834762	N.D.
mmu-miR-137-3p	4.76E-05	5.834762	N.D.
mmu-miR-30a-3p	9.94E-05	5.768126	N.D.

mmu-miR-30a-3p	9.94E-05	5.768126	N.D.
mmu-miR-30d-5p	3.17E-05	7.684248	N.D.
mmu-miR-129-2-3p	2.8E-05	6.09428	N.D.
mmu-miR-181a-1-3p	4.97E-05	5.956292333	N.D.
mmu-miR-181a-1-3p	4.97E-05	5.956292333	N.D.
mmu-miR-543-3p	0.000121	5.961046	N.D.
mmu-miR-425-5p	3.26E-05	6.538779	N.D.
mmu-miR-434-5p	2.64E-05	5.451562	N.D.
mmu-miR-434-5p	2.64E-05	5.451562	N.D.
mmu-miR-5100	5.23E-05	7.093260667	N.D.
mmu-miR-329-3p	0.000318	6.487526333	N.D.
mmu-miR-1983	6.66E-05	5.696977333	N.D.
mmu-miR-212-3p	5.16E-05	6.409489333	N.D.
mmu-miR-20b-5p	0.000121	6.508302	N.D.
mmu-miR-376b-3p	2.45E-05	6.167027	N.D.
mmu-miR-301a-3p	9.44E-05	5.213394333	N.D.
mmu-miR-31-5p	3.17E-05	6.172028667	N.D.
mmu-miR-31-5p	3.17E-05	6.172028667	N.D.
mmu-miR-326-3p	9.37E-05	6.267412667	N.D.
mmu-miR-326-3p	9.37E-05	6.267412667	N.D.
mmu-miR-195a-5p	0.000381	7.461465333	N.D.
mmu-miR-27b-3p	0.000183	6.112958667	N.D.
mmu-miR-300-3p	3.17E-05	6.772393667	N.D.
mmu-miR-187-3p	0.00012	6.376167333	N.D.
mmu-miR-770-5p	3.17E-05	6.570839333	N.D.
mmu-miR-770-5p	3.17E-05	6.570839333	N.D.
mmu-miR-138-2-3p	0.000207	5.063075	N.D.
mmu-miR-138-2-3p	0.000207	5.063075	N.D.
mmu-miR-409-5p	8.46E-05	6.194656333	N.D.
mmu-miR-325-5p	0.000164	4.714664	N.D.
mmu-miR-325-5p	0.000164	4.714664	N.D.
mmu-miR-335-5p	0.000183	5.221403333	N.D.
mmu-miR-3475-3p	0.000126	5.872268333	N.D.
mmu-miR-17-3p	4.48E-05	7.209764667	N.D.
mmu-miR-28a-5p	0.000524	5.035764333	N.D.
mmu-miR-421-3p	0.000498	5.205851	N.D.
mmu-miR-193b-3p	5.06E-05	6.772432667	N.D.
mmu-miR-322-3p	4.48E-05	4.843634	N.D.
mmu-miR-322-3p	4.48E-05	4.843634	N.D.
mmu-miR-505-5p	9.21E-05	6.780423667	N.D.
mmu-miR-29a-3p	4.76E-05	4.71236	N.D.
mmu-miR-1839-3p	7.39E-05	4.504895667	N.D.
mmu-miR-1839-3p	7.39E-05	4.504895667	N.D.
mmu-miR-350-3p	0.000162	4.788322	N.D.
mmu-miR-339-5p	6.14E-05	5.301924333	N.D.
mmu-miR-339-5p	6.14E-05	5.301924333	N.D.
mmu-miR-378a-3p	0.000192	5.023367667	N.D.

mmu-miR-27a-3p	0.000242	5.073536667	N.D.
mmu-miR-27a-3p	0.000242	5.073536667	N.D.
mmu-miR-411-5p	5.06E-05	5.313449333	N.D.
mmu-miR-345-5p	4.76E-05	6.664290333	N.D.
mmu-miR-124-5p	5.56E-05	5.958355333	N.D.
mmu-miR-124-5p	5.56E-05	5.958355333	N.D.
mmu-miR-152-3p	0.0028	4.857485667	N.D.
mmu-miR-500-3p	4.76E-05	6.822108667	N.D.
mmu-miR-485-5p	5.16E-05	6.347045667	N.D.
mmu-miR-30c-2-3p	0.000124	5.541818333	N.D.
mmu-miR-369-5p	0.000713	4.659703333	N.D.
mmu-miR-369-5p	0.000713	4.659703333	N.D.
mmu-miR-126a-3p	0.000161	4.744896	N.D.
mmu-miR-126a-3p	0.000161	4.744896	N.D.
mmu-miR-127-5p	4.97E-05	5.135600667	N.D.
mmu-miR-7a-1-3p	0.00359	4.076884667	N.D.
mmu-miR-7a-1-3p	0.00359	4.076884667	N.D.
mmu-miR-323-3p	0.002804	4.363317667	N.D.
mmu-miR-323-3p	0.002804	4.363317667	N.D.
mmu-miR-485-3p	4.76E-05	5.777509	N.D.
mmu-miR-381-3p	0.000197	5.027466667	N.D.
mmu-miR-495-3p	0.000431	5.472258	N.D.
mmu-miR-497-5p	0.000324	4.732701667	N.D.
mmu-miR-497-5p	0.000324	4.732701667	N.D.
mmu-miR-154-5p	7.39E-05	5.201245333	N.D.
mmu-miR-344d-3p	0.000271	4.434687	N.D.
mmu-miR-344d-3p	0.000271	4.434687	N.D.
mmu-miR-324-5p	7.39E-05	6.425102667	N.D.
mmu-miR-411-3p	0.000484	4.353647667	N.D.
mmu-miR-337-5p	4.42E-05	5.806225667	N.D.
mmu-miR-410-3p	7.39E-05	4.335804667	N.D.
mmu-miR-423-3p	9.93E-05	6.394651333	N.D.
mmu-miR-423-3p	9.93E-05	6.394651333	N.D.
mmu-miR-138-1-3p	0.000228	5.040263	N.D.
mmu-miR-138-1-3p	0.000228	5.040263	N.D.
mmu-miR-182-5p	0.006285	4.539657667	N.D.
mmu-miR-182-5p	0.006285	4.539657667	N.D.
mmu-miR-412-5p	0.000132	4.275014333	N.D.
mmu-miR-412-5p	0.000132	4.275014333	N.D.
mmu-miR-30e-5p	0.002829	3.976267333	N.D.
mmu-miR-30e-5p	0.002829	3.976267333	N.D.
mmu-miR-301b-3p	0.000183	4.310233667	N.D.
mmu-miR-3102-3p	0.000311	6.094772	N.D.
mmu-miR-192-5p	0.022181	3.399199667	N.D.
mmu-miR-192-5p	0.022181	3.399199667	N.D.
mmu-miR-467a-5p	0.006975	4.043578	N.D.
mmu-miR-467a-5p	0.006975	4.043578	N.D.

mmu-miR-125b-2-3p	0.000461	4.35888	N.D.
mmu-miR-1949	0.002197	5.117966667	N.D.
mmu-miR-146b-5p	0.02868	3.351785667	N.D.
mmu-miR-146b-5p	0.02868	3.351785667	N.D.
mmu-miR-449a-5p	0.03386	3.747559333	N.D.
mmu-miR-30e-3p	0.019606	3.263114667	N.D.
mmu-miR-30e-3p	0.019606	3.263114667	N.D.
mmu-miR-673-5p	0.043088	3.768979333	N.D.
mmu-miR-540-3p	0.000202	4.687803	N.D.
mmu-miR-540-3p	0.000202	4.687803	N.D.
mmu-miR-122-5p	0.002862	4.052558	N.D.
mmu-miR-551b-3p	0.005773	3.395753333	N.D.
mmu-miR-551b-3p	0.005773	3.395753333	N.D.
mmu-miR-28a-3p	0.004948	4.807170667	N.D.
mmu-miR-140-5p	0.018715	3.302621	N.D.
mmu-miR-6239	0.000242	5.161998333	N.D.
mmu-miR-504-5p	0.000485	4.06877	N.D.
mmu-miR-504-5p	0.000485	4.06877	N.D.
mmu-miR-380-5p	0.027503	3.582742667	N.D.
mmu-miR-376a-3p	0.000131	3.196005667	N.D.
mmu-miR-7b-3p	0.001401	4.891506	N.D.
mmu-miR-6944-3p	0.009955	3.163255667	N.D.
mmu-miR-135a-2-3p	0.008909	4.570320333	N.D.
mmu-miR-212-5p	0.018807	3.255250667	N.D.
mmu-miR-212-5p	0.018807	3.255250667	N.D.
mmu-miR-210-3p	0.002304	4.980513667	N.D.
mmu-miR-210-3p	0.002304	4.980513667	N.D.
mmu-miR-1969	0.025667	3.097878667	N.D.
mmu-miR-34a-5p	0.001918	4.623536333	N.D.
mmu-miR-431-3p	0.013284	4.501277333	N.D.
mmu-miR-431-3p	0.013284	4.501277333	N.D.
mmu-miR-6367	0.00529	3.835214	N.D.

Supplementary Table 2
miRs that are enriched in neurons. N: neurons; E: exosomes;

miR ID	FC (N/E)	FDR q	Expression in N (Log_2)	Expression in E (Log_2)
mmu-miR-709	9.27668392	7.39E-05	13.58274667	10.35653
mmu-miR-124-3p	6.007021574	0.000183	12.79306333	10.22079
mmu-miR-16-5p	5.067848062	0.000246	11.15737	8.82717
mmu-miR-26a-5p	11.89898408	0.000245	12.07892667	8.5771765
mmu-miR-342-3p	8.668971405	0.000106	11.29528	8.189725
mmu-miR-125a-5p	7.616670102	0.000323	11.08431	8.1226795
mmu-miR-103-3p	14.03059968	0.00013	11.95626	8.057432
mmu-let-7d-5p	22.21392841	4.27E-05	12.18826	7.7189365
mmu-let-7a-5p	14.68348585	5.35E-05	11.33612	7.464382
mmu-miR-191-5p	10.51118405	6.33E-05	10.7246	7.3443815
mmu-miR-17-5p	13.59907452	7.77E-05	10.98836	7.2555615
mmu-miR-107-3p	21.67305548	3.17E-05	11.36268667	6.893959
mmu-miR-6240	5.971598917	0.004078	9.561862	6.715648
mmu-miR-674-5p	5.060337512	0.000431	9.002087667	6.638081
mmu-miR-690	21.48698013	6.66E-05	10.874	6.3960485
mmu-miR-128-3p	14.20683934	0.000228	10.17381667	6.21904
mmu-miR-93-5p	16.26417622	8.46E-05	10.28173667	6.200796
mmu-miR-181b-5p	20.65067917	4.76E-05	10.47778333	6.120649
mmu-miR-185-5p	9.293002342	5.06E-05	9.292852	6.079353
mmu-miR-99b-5p	29.20514836	2.8E-05	10.62202	5.772895
mmu-miR-3099-3p	5.360468859	0.000161	8.088104333	5.6624985
mmu-let-7e-5p	59.14801998	3.17E-05	11.50356333	5.5236665
mmu-miR-370-3p	5.10808231	0.000156	7.874027333	5.522815
mmu-miR-433-3p	10.0404524	0.000119	8.872084333	5.4977245
mmu-miR-361-5p	17.04012304	5.06E-05	9.552695333	5.4825375
mmu-miR-92b-3p	8.857958997	0.000261	8.503176	5.268499
mmu-miR-181a-5p	105.219624	1.56E-05	11.61656667	4.9006225
mmu-miR-15b-5p	11.54082031	0.000139	8.278603	4.7910305
mmu-miR-20a-5p	32.69890742	9.29E-05	9.843220667	4.7795115
mmu-miR-23a-3p	16.80973915	0.000212	8.773478	4.644608
mmu-miR-25-3p	6.103269076	0.002109	7.312611333	4.6226175
mmu-miR-149-5p	25.42267746	9.29E-05	9.391677333	4.5913915
mmu-miR-134-5p	5.223128285	0.000155	6.967403	4.5848535
mmu-miR-541-5p	24.25142996	2.54E-05	9.070993	4.4700575
mmu-miR-6540-5p	10.17420783	5.16E-05	7.729673333	4.386302
mmu-miR-106a-5p	21.58969212	0.000102	8.683444333	4.298389
mmu-miR-138-5p	25.71010922	2.8E-05	8.896216333	4.217492
mmu-miR-140-3p	13.54306346	0.000125	7.956660333	4.20158
mmu-miR-652-3p	17.31776056	4.48E-05	8.301484	4.168928
mmu-miR-125b-1-3p	8.718261541	0.000318	7.339206333	4.1515835
mmu-miR-1981-5p	4.412985093	0.000479	6.218971667	4.0698235
mmu-miR-328-3p	8.381687911	0.001421	7.289296667	3.9709875

mmu-miR-342-5p	13.53779261	0.000242	7.848695667	3.955616
mmu-miR-181d-5p	18.26068014	6.6E-05	7.995192	3.8428475
mmu-miR-106b-3p	9.643311674	0.00037	7.210592	3.805636
mmu-miR-668-3p	13.55046371	4.97E-05	7.547530333	3.7693925
mmu-miR-668-3p	13.55046371	4.97E-05	7.547530333	3.7693925
mmu-miR-324-3p	4.529216195	0.001155	5.993108333	3.75652
mmu-miR-106b-5p	110.0469305	3.98E-05	10.41472433	3.668777
mmu-miR-501-3p	11.50198941	5.06E-05	7.138326667	3.620825
mmu-miR-130a-3p	20.68764241	5.06E-05	7.929543667	3.579312
mmu-miR-99a-5p	120.8397487	1.56E-05	10.45669	3.557304
mmu-miR-351-5p	9.375927127	9.15E-05	6.721294667	3.5106045
mmu-miR-351-5p	9.375927127	9.15E-05	6.721294667	3.5106045
mmu-miR-532-5p	18.55967367	2.8E-05	7.715155333	3.506096
mmu-miR-128-2-5p	5.441698921	0.000412	5.967864667	3.4794655
mmu-miR-1231-5p	5.065225539	0.000463	5.814554667	3.4669615
mmu-miR-487b-3p	75.61555454	1.56E-05	9.710969333	3.4612185
mmu-miR-409-3p	13.32168291	5.59E-05	7.1776	3.461097
mmu-miR-127-3p	57.3919012	1.56E-05	9.302446333	3.451138
mmu-miR-484	5.085989545	0.001084	5.845128333	3.4244825
mmu-let-7g-5p	22.06574833	0.000492	8.066514667	3.367507
mmu-miR-125a-3p	5.665127858	0.000243	5.831001333	3.3339035
mmu-miR-222-3p	21.24138044	2.45E-05	7.726799333	3.317334
mmu-miR-151-3p	14.24807496	6.67E-05	7.088755	3.250379
mmu-miR-1839-5p	14.44062787	0.000152	7.049208	3.246696
mmu-miR-674-3p	9.603180665	7.39E-05	6.467302	3.2179015
mmu-miR-532-3p	16.59872574	4.25E-05	7.216212	3.170494
mmu-miR-330-3p	9.643858214	6.81E-05	6.438905667	3.162076
mmu-let-7k	9.537230917	0.000204	6.468795333	3.147179
mmu-miR-345-3p	8.075617879	9.74E-05	6.143040333	3.1434035
mmu-miR-100-5p	68.15241533	3.17E-05	9.221762333	3.132121
mmu-miR-708-5p	66.07648633	3.98E-05	9.273014667	3.119697
mmu-miR-486-5p	9.799858352	9.44E-05	6.411383667	3.1093915
mmu-miR-3107-5p	9.799858352	9.44E-05	6.411383667	3.1093915
mmu-miR-494-3p	7.353746643	0.00012	5.943327	3.0722385
mmu-miR-221-3p	20.95422595	3.17E-05	7.448659	3.045896
mmu-miR-676-3p	24.8766971	4.76E-05	7.699927333	3.037066

Supplementary Table 3
miRs selectively expressed in neuronal exosomes. N: neurons; E: exosome; N.D.: not detected;

miR ID	FDR q	Expression in E (Log_2)	Expression in N
mmu-miR-6988-5p	0.007358	4.8257965	N.D.
mmu-miR-5620-5p	0.000295	6.8389805	N.D.
mmu-miR-6907-5p	0.001918	5.016434	N.D.
mmu-miR-8095	0.005048	6.9059545	N.D.
mmu-miR-5135	0.00093	5.8512015	N.D.
mmu-miR-669l-5p	0.000353	8.339154	N.D.
mmu-miR-466i-5p	0.004521	6.109399	N.D.
mmu-miR-669e-5p	5.61E-05	8.780531	N.D.
mmu-miR-669n	9.67E-05	7.249937	N.D.
mmu-miR-5620-3p	0.001278	6.651835	N.D.
mmu-miR-6956-5p	0.005469	5.078461	N.D.
mmu-miR-669d-5p	0.001302	8.3668625	N.D.
mmu-miR-6405	0.004948	4.8785045	N.D.
mmu-miR-7682-3p	0.000713	6.2697165	N.D.
mmu-miR-467h	0.002497	7.4303695	N.D.
mmu-miR-7231-5p	0.002495	5.5361685	N.D.
mmu-miR-6378	0.002486	6.179964	N.D.
mmu-miR-3473d	0.004591	4.9697665	N.D.
mmu-miR-3473d	0.004591	4.9697665	N.D.
mmu-miR-5129-3p	0.036149	4.4619885	N.D.
mmu-miR-493-3p	0.002071	5.0535245	N.D.
mmu-miR-7014-5p	0.000937	4.2516855	N.D.
mmu-miR-6409	0.000103	6.160503	N.D.
mmu-miR-6337	0.001401	5.7119685	N.D.
mmu-miR-468-3p	0.003471	5.8381105	N.D.
mmu-miR-7034-5p	0.001599	4.7703205	N.D.
mmu-miR-7058-5p	0.002426	5.000894	N.D.
mmu-miR-6985-5p	0.000134	5.0635885	N.D.
mmu-miR-6979-5p	0.00445	4.103257	N.D.
mmu-miR-3569-5p	0.002071	4.2605755	N.D.
mmu-miR-698-5p	0.002174	4.5857675	N.D.
mmu-miR-7082-3p	0.012051	5.372015	N.D.
mmu-miR-7075-5p	8.46E-05	4.7204075	N.D.
mmu-miR-3470b	0.000385	4.0628465	N.D.
mmu-miR-194-2-3p	0.00108	4.1078315	N.D.
mmu-miR-7087-5p	0.000738	4.5783585	N.D.
mmu-miR-7666-5p	0.000202	5.4703065	N.D.
mmu-miR-7666-5p	0.000202	5.4703065	N.D.
mmu-miR-365-2-5p	0.006421	4.0009515	N.D.
mmu-miR-3092-3p	0.002156	5.021361	N.D.
mmu-miR-3064-5p	0.00093	4.6267695	N.D.
mmu-miR-6911-5p	0.000155	4.9807745	N.D.

mmu-miR-6241	0.000202	4.25387	N.D.
mmu-miR-16-2-3p	0.004344	3.988401	N.D.
mmu-miR-7059-5p	0.002109	4.0467695	N.D.
mmu-miR-214-5p	0.001727	4.5676195	N.D.
mmu-miR-185-3p	0.013041	3.5527285	N.D.
mmu-miR-1971	0.008554	4.4085845	N.D.
mmu-miR-7212-5p	0.022464	4.422684	N.D.
mmu-miR-7046-3p	0.006086	3.181118	N.D.
mmu-miR-341-3p	0.001013	4.300059	N.D.
mmu-miR-341-3p	0.001013	4.300059	N.D.
mmu-miR-680	0.004761	3.805039	N.D.
mmu-miR-680	0.004761	3.805039	N.D.
mmu-miR-7038-5p	0.000655	5.2696255	N.D.
mmu-miR-6942-5p	0.000234	4.917974	N.D.
mmu-miR-7015-5p	0.002071	3.990892	N.D.
mmu-miR-7008-5p	0.00024	4.8613945	N.D.
mmu-miR-7008-5p	0.00024	4.8613945	N.D.
mmu-miR-3075-5p	0.000621	4.288436	N.D.
mmu-miR-296-5p	0.000515	5.012217	N.D.
mmu-miR-296-5p	0.000515	5.012217	N.D.
mmu-miR-327	0.000718	3.8805245	N.D.
mmu-miR-6958-5p	0.000393	5.714901	N.D.
mmu-miR-6929-3p	0.000126	5.1258435	N.D.
mmu-miR-7024-5p	0.000264	5.1196525	N.D.
mmu-miR-3098-3p	8.38E-05	4.878502	N.D.
mmu-miR-7211-3p	0.000909	3.829621	N.D.
mmu-miR-7211-3p	0.000909	3.829621	N.D.
mmu-miR-7216-5p	0.000264	4.111371	N.D.
mmu-miR-1951	0.000462	3.8896815	N.D.
mmu-miR-7004-5p	6.14E-05	5.788396	N.D.
mmu-miR-6959-5p	0.000155	3.981328	N.D.
mmu-miR-6959-5p	0.000155	3.981328	N.D.
mmu-miR-6998-5p	0.002267	4.419864	N.D.
mmu-miR-365-1-5p	8.46E-05	4.7534175	N.D.
mmu-miR-1249-3p	0.00013	4.036608	N.D.
mmu-miR-1249-3p	0.00013	4.036608	N.D.
mmu-miR-704	0.002424	3.2036995	N.D.
mmu-miR-451b	0.000498	3.653444	N.D.
mmu-miR-6418-5p	0.000655	3.871236	N.D.
mmu-miR-1967	0.001698	3.352441	N.D.
mmu-miR-483-3p	0.001682	3.910792	N.D.
mmu-miR-32-3p	0.000106	3.8697635	N.D.
mmu-miR-7051-3p	0.001069	4.1768085	N.D.
mmu-miR-7051-3p	0.001069	4.1768085	N.D.
mmu-miR-6902-3p	0.000459	3.397917	N.D.
mmu-miR-6902-3p	0.000459	3.397917	N.D.
mmu-miR-7119-5p	0.000192	3.967256	N.D.

mmu-miR-8108	0.00022	4.006416	N.D.
mmu-miR-6967-5p	0.000704	3.4545185	N.D.
mmu-miR-3109-5p	0.002299	4.154368	N.D.
mmu-miR-3109-5p	0.002299	4.154368	N.D.
mmu-miR-743a-5p	0.000161	4.238502	N.D.
mmu-miR-297c-5p	0.007691	4.2604415	N.D.

Supplementary Table 4
miRs that are enriched in neuronal exosomes. N: neurons; E: exosomes;

miR ID	FC (E/N)	FDR q	Expression in N (Log_2)	Expression in E (Log_2)
mmu-miR-8101	8.194992266	0.000183	8.949468	11.978345
mmu-miR-5130	4.527710163	0.000716	9.300458667	11.49509
mmu-miR-669m-5p	38.9561074	2.41E-05	5.799245	11.08653
mmu-miR-466m-5p	38.9561074	2.41E-05	5.799245	11.08653
mmu-miR-6366	6.536558004	0.00108	8.427901667	11.04273
mmu-miR-669f-5p	36.78006455	4.48E-05	5.630052333	10.884365
mmu-miR-466j	45.47252797	6.14E-05	5.173512333	10.796515
mmu-miR-195a-3p	12.60707338	0.000171	7.022227333	10.71868
mmu-miR-6931-5p	8.237276503	6.67E-05	7.554615	10.600145
mmu-miR-466f	35.34226866	2.54E-05	5.15698	10.322315
mmu-miR-466f-5p	32.37255855	9.37E-05	4.968492	10.0953205
mmu-miR-297a-5p	38.52343198	4.48E-05	4.676119333	9.993628
mmu-miR-7030-5p	9.909199146	0.000102	6.381194	9.7179285
mmu-miR-7118-5p	5.379462053	0.000269	7.300005333	9.717679
mmu-miR-669b-5p	36.09887791	0.000124	4.197621	9.529075
mmu-miR-16-1-3p	8.248084914	0.000286	6.330563	9.400988
mmu-miR-1892	5.861211613	0.000355	6.522041667	9.0441465
mmu-miR-466h-5p	24.11925127	0.000156	4.293115333	9.004549
mmu-miR-3082-5p	25.42970686	0.000245	4.412145	8.998149
mmu-miR-711	5.752018117	0.000419	6.511639667	8.9946925
mmu-miR-574-5p	24.09160966	0.000234	4.157375667	8.7742345
mmu-miR-1187	29.51731619	0.000856	3.314362667	8.597365
mmu-miR-466c-5p	32.2289029	2.8E-05	3.563531	8.5845205
mmu-miR-669k-5p	25.13724078	8.46E-05	3.665043	8.317232
mmu-miR-669o-5p	30.58555661	0.000323	3.196016333	8.1253525
mmu-miR-669a-5p	18.48827176	0.001265	3.726311667	7.5889425
mmu-miR-669p-5p	18.48827176	0.001265	3.726311667	7.5889425
mmu-miR-7666-3p	5.106909409	0.004861	5.192038667	7.481102
mmu-miR-669c-5p	9.54645238	0.000966	4.080145	7.4782515
mmu-miR-1930-3p	19.34579808	0.000196	3.120183	7.376518
mmu-miR-696	14.210697	0.000693	3.402310667	7.161024
mmu-miR-15a-3p	8.466263082	0.011833	3.4915	7.1036715
mmu-miR-665-5p	10.15424936	0.014621	3.430532	7.0241115
mmu-miR-7222-3p	7.216176204	0.004817	3.822829667	6.568517
mmu-miR-7003-5p	8.417924092	0.004376	3.431139333	6.512956
mmu-miR-8102	4.033463152	0.00136	4.488872333	6.469205
mmu-miR-1982-5p	5.125557067	0.003499	3.913565	6.4090055
mmu-miR-6910-5p	4.179979422	0.002446	4.205555	6.3275535
mmu-miR-6391	9.171811612	0.000344	3.030877667	6.3020595
mmu-miR-5622-3p	9.240209744	0.005922	3.162617667	6.217842
mmu-miR-6908-5p	4.606293917	0.000305	3.651349667	5.864621
mmu-miR-7048-5p	4.432093437	0.004511	3.538935667	5.827218

mmu-miR-6941-5p	5.108193868	0.009017	3.161399333	5.7625565
mmu-miR-7684-3p	5.091598612	0.011534	3.007451	5.620581
mmu-miR-702-5p	4.329963479	0.002025	3.480794667	5.5972335
mmu-miR-7005-5p	4.828274565	0.002364	3.03278	5.414988
mmu-miR-7672-5p	4.391096825	0.003375	3.081087667	5.324648

Supplementary Table 5

miRs highly expressed in both neurons and neuronal exosomes. N: neurons; E: exosomes;

miR ID	FC	FDR q	Expression in N (Log_2)	Expression in E (Log_2)
mmu-miR-2137	3.549928	0.000621	12.27007	14.10373
mmu-miR-3960	1.703952	0.026513	11.63930667	12.40456
mmu-miR-149-3p	1.837432	0.016946	11.33383	12.222275
mmu-let-7c-5p	2.83611	0.00233	13.58675	12.091895
mmu-miR-8101	8.194992	0.000183	8.949468	11.978345
mmu-let-7b-5p	2.814354	0.002446	13.08052667	11.565825
mmu-miR-5130	4.52771	0.000716	9.300458667	11.49509
mmu-miR-125b-5p	3.158873	0.003375	13.08679667	11.45695
mmu-miR-762	2.575935	0.046272	9.866065667	11.08404
mmu-miR-6366	6.536558	0.00108	8.427901667	11.04273
mmu-miR-8110	1.908444	0.030601	9.676484667	10.57575
mmu-miR-709	9.276684	7.39E-05	13.58274667	10.35653
mmu-miR-124-3p	6.007022	0.000183	12.79306333	10.22079
mmu-miR-3547-5p	2.057596	0.011051	9.157264333	10.2171
mmu-miR-7045-5p	1.749488	0.030867	9.295351	10.115008
mmu-miR-5128	2.419677	0.003321	8.504018667	9.7761915
mmu-miR-24-3p	1.774725	0.036149	9.756437	8.9048005
mmu-miR-16-5p	5.067848	0.000246	11.15737	8.82717
mmu-miR-26a-5p	11.89898	0.000245	12.07892667	8.5771765
mmu-let-7i-5p	2.21031	0.025938	9.599434	8.4590275
mmu-miR-320-3p	2.765689	0.002828	9.822620667	8.3335795
mmu-miR-342-3p	8.668971	0.000106	11.29528	8.189725
mmu-miR-125a-5p	7.61667	0.000323	11.08431	8.1226795
mmu-miR-103-3p	14.0306	0.00013	11.95626	8.057432
