# Supplementary material for: Exosome reporter mice reveal the involvement of exosomes in mediating neuron to astroglia communication in the CNS
Source: Nat Commun. 2019 Sep 12;10:4136. doi: 10.1038/s41467-019-11534-w (PMC6742670; doi:10.1038/s41467-019-11534-w)
Supplement: Supplementary file 2 — Reporting Summary [file 41467_2019_11534_MOESM2_ESM.pdf]

nature research

Corresponding author(s): Yongjie Yang
Last updated by author(s): June, 27th, 2019

Reporting Summary

Nature Research wishes to improve the reproducibility of the work that we publish. This form provides structure for consistency and transparency in reporting. For further information on Nature Research policies, see Authors & Referees and the Editorial Policy Checklist.

Statistics

For all statistical analyses, confirm that the following items are present in the figure legend, table legend, main text, or Methods section.

n/a	Confirmed	
☐	☒	The exact sample size (*n*) for each experimental group/condition, given as a discrete number and unit of measurement
☐	☒	A statement on whether measurements were taken from distinct samples or whether the same sample was measured repeatedly
☐	☒	The statistical test(s) used AND whether they are one- or two-sided<br>*Only common tests should be described solely by name; describe more complex techniques in the Methods section.*
☒	☐	A description of all covariates tested
☐	☒	A description of any assumptions or corrections, such as tests of normality and adjustment for multiple comparisons
☒	☐	A full description of the statistical parameters including central tendency (e.g. means) or other basic estimates (e.g. regression coefficient) AND variation (e.g. standard deviation) or associated estimates of uncertainty (e.g. confidence intervals)
☐	☒	For null hypothesis testing, the test statistic (e.g. *F*, *t*, *r*) with confidence intervals, effect sizes, degrees of freedom and *P* value noted<br>*Give P values as exact values whenever suitable.*
☒	☐	For Bayesian analysis, information on the choice of priors and Markov chain Monte Carlo settings
☒	☐	For hierarchical and complex designs, identification of the appropriate level for tests and full reporting of outcomes
☐	☒	Estimates of effect sizes (e.g. Cohen's *d*, Pearson's *r*), indicating how they were calculated

Our web collection on statistics for biologists contains articles on many of the points above.

Software and code

Policy information about availability of computer code

Data collection	Izon qNano software (version 3.2); Nikon NIS-Elements; Leica LAS X; ChemiDoc imaging software; Summit version 4.3 software
Data analysis	Affymetrix Expression Console (version 1.3.0.187); Imaris imaging software (version 7.7.1); Image J (Fuji); Prism 7;

For manuscripts utilizing custom algorithms or software that are central to the research but not yet described in published literature, software must be made available to editors/reviewers. We strongly encourage code deposition in a community repository (e.g. GitHub). See the Nature Research guidelines for submitting code & software for further information.

Data

Policy information about availability of data

All manuscripts must include a data availability statement. This statement should provide the following information, where applicable:
- Accession codes, unique identifiers, or web links for publicly available datasets
- A list of figures that have associated raw data
- A description of any restrictions on data availability

No large datasets were generated from this study. The data that support the findings of this study are from the corresponding author upon reasonable request.

Field-specific reporting

Please select the one below that is the best fit for your research. If you are not sure, read the appropriate sections before making your selection.

☒ Life sciences ☐ Behavioural & social sciences ☐ Ecological, evolutionary & environmental sciences

For a reference copy of the document with all sections, see nature.com/documents/nr-reporting-summary-flat.pdf

Life sciences study design

All studies must disclose on these points even when the disclosure is negative.

Sample size	Sample size for each experiment is described in each method section and in figure legends. Previous results and the G*Power (version 3.1) were used to estimate the reasonable sample size.
Data exclusions	Fig. 8d, the 132 A/S sample, the interquartile range approach was used to exclude outliers.
Replication	Our current study generated a new mouse tool from which the experimental outcome can be reliably observed. We have been using reagents that have been verified. In addition, we have repeated all experimental findings sufficiently, as described in the experimental design and figure legend.
Randomization	For mice experiments, gender was considered and randomized with similar number in each group. For in vitro experiments, control and experimental groups are always randomly assigned.
Blinding	Mice experiments were blindly performed. In vitro experiments were not blind, but all controls were included. Quantification was done blindly.

Reporting for specific materials, systems and methods

We require information from authors about some types of materials, experimental systems and methods used in many studies. Here, indicate whether each material, system or method listed is relevant to your study. If you are not sure if a list item applies to your research, read the appropriate section before selecting a response.

Materials & experimental systems

n/a	Involved in the study
☐	☒ Antibodies
☒	☐ Eukaryotic cell lines
☒	☐ Palaeontology
☐	☒ Animals and other organisms
☒	☐ Human research participants
☒	☐ Clinical data

Methods

n/a	Involved in the study
☒	☐ ChIP-seq
☐	☒ Flow cytometry
☒	☐ MRI-based neuroimaging

Antibodies

Antibodies used	All antibodies used in the study has been clearly described in the materials and methods, including the catalog number and vendor.
Validation	All antibodies have been either commercially validated or validated in the literature, or in our study.

Animals and other organisms

Policy information about studies involving animals; ARRIVE guidelines recommended for reporting animal research

Laboratory animals	Mice were used. All details were described in the materials and methods.
Wild animals	N/A.
Field-collected samples	N/A.
Ethics oversight	Tufts Tufts University IACUC.

Note that full information on the approval of the study protocol must also be provided in the manuscript.

Flow Cytometry

Plots

Confirm that:

- [ ] The axis labels state the marker and fluorochrome used (e.g. CD4-FITC).
- [ ] The axis scales are clearly visible. Include numbers along axes only for bottom left plot of group (a 'group' is an analysis of identical markers).
- [ ] All plots are contour plots with outliers or pseudocolor plots.
- [ ] A numerical value for number of cells or percentage (with statistics) is provided.

Methodology

Sample preparation	See details in the materials and methods
Instrument	MoFlo MLS high speed cell sorter (Beckman Coulter)
Software	Summit version 4.3 software
Cell population abundance	The sorting is based on eGFP fluorescence driven by the astrocyte specific aldh1l1 promoter, therefore the population is highly abundant.
Gating strategy	based on the eGFP fluorescence intensity. The positive population is clearly distinct (> 100 fold brighter) than the wild type control population.

- [ ] Tick this box to confirm that a figure exemplifying the gating strategy is provided in the Supplementary Information.
